# Supplementary material for: Unconventional Targeting of a Thiol Peroxidase to the Mitochondrial Intermembrane Space Facilitates Oxidative Protein Folding
Source: Cell Rep. 2017 Mar 14;18(11):2729–41. doi: 10.1016/j.celrep.2017.02.053 (PMC5368413; doi:10.1016/j.celrep.2017.02.053)
Supplement: Document S1. Supplemental Experimental Procedures and Figures S1–S5 [file mmc1.pdf]

Cell Reports, Volume *18*

Supplemental Information

Unconventional Targeting of a Thiol Peroxidase to the Mitochondrial Intermembrane Space Facilitates Oxidative Protein Folding

Paraskevi Kritsiligkou, Afroditi Chatzi, Georgia Charalampous, Aleksandr Mironov, Jr., Chris M. Grant, and Kostas Tokatlidis

Unconventional targeting of a thiol peroxidase to the mitochondrial intermembrane space facilitates oxidative protein folding

Paraskevi Kritsiligkou[1,3], Afroditi Chatzi[2,3], Georgia Charalampous[2], Aleksandr Mironov Jr.[1], Chris M. Grant[1,4], and Kostas Tokatlidis[2,4]

SUPPLEMENTARY MATERIAL

[1] Faculty of Biology, Medicine and Health, University of Manchester, Manchester, M13 9PL, UK

[2] Institute of Molecular, Cell and Systems Biology, College of Medical, Veterinary and Life Sciences, University of Glasgow, Glasgow, G12 8QQ, UK

[3] Co-first authors

[4] Co-senior authors

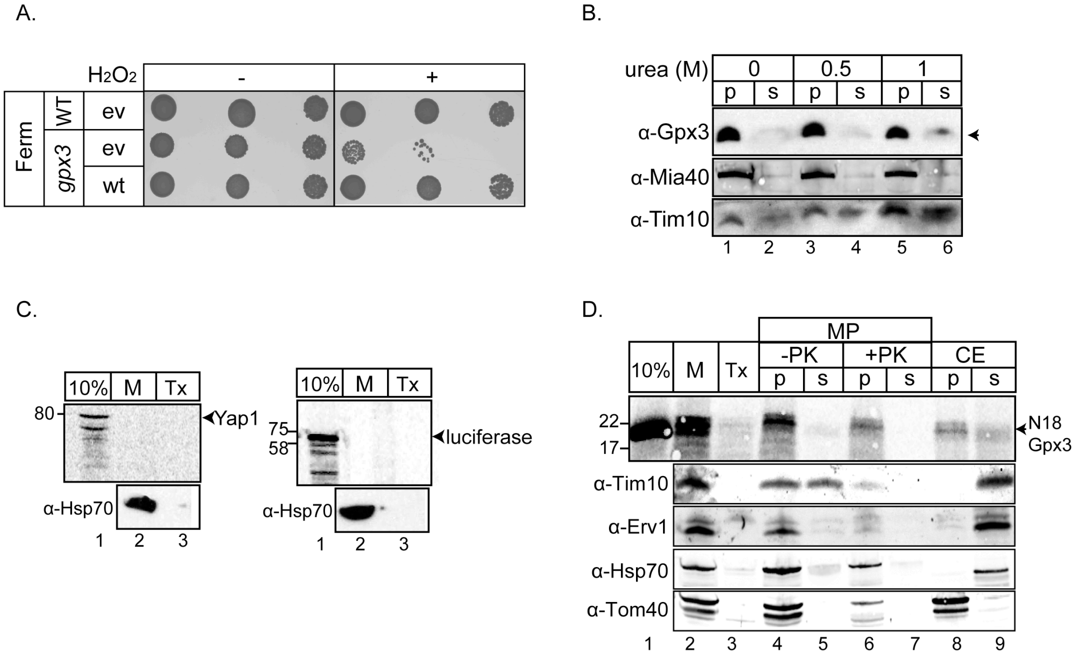

Figure S1, related to Figure 1: A) Gpx3 expressed myc-tagged from a plasmid can rescue the phenotype of a *gpx3* upon H2O2 stress similar to WT levels. Wt or *gpx3* strains were transformed with empty vector (ev) or WT (Gpx3-myc) plasmid. Cells were spotted on SCD (Ferm) plates with or without H_2O_2.
B) WT mitochondria were incubated in SEH buffer (0.6 M sorbitol, 5 mM EDTA, 20 mM Hepes pH 7.4) containing different concentrations of urea for 10 min at 30°C. Mitochondria were isolated via centrifugation and the supernatant was TCA precipitated prior to SDS analysis and immunodecoration against proteins of interest. C) The radioactive precursors Yap1 and luciferase were prepared in the same conditions as Gpx3 and import assays in WT mitochondria were performed. D) Import of radiolabeled N18Gpx3 in WT mitochondria 20min (M) (autoradiography). To verify the specificity of the import, samples were also treated with TritonX before exposure to protease (Tx). Further fractionation was performed, using osmotic shock in the presence or absence of protease (MP samples). Finally the soluble proteins were also obtained by carbonate extraction (CE).

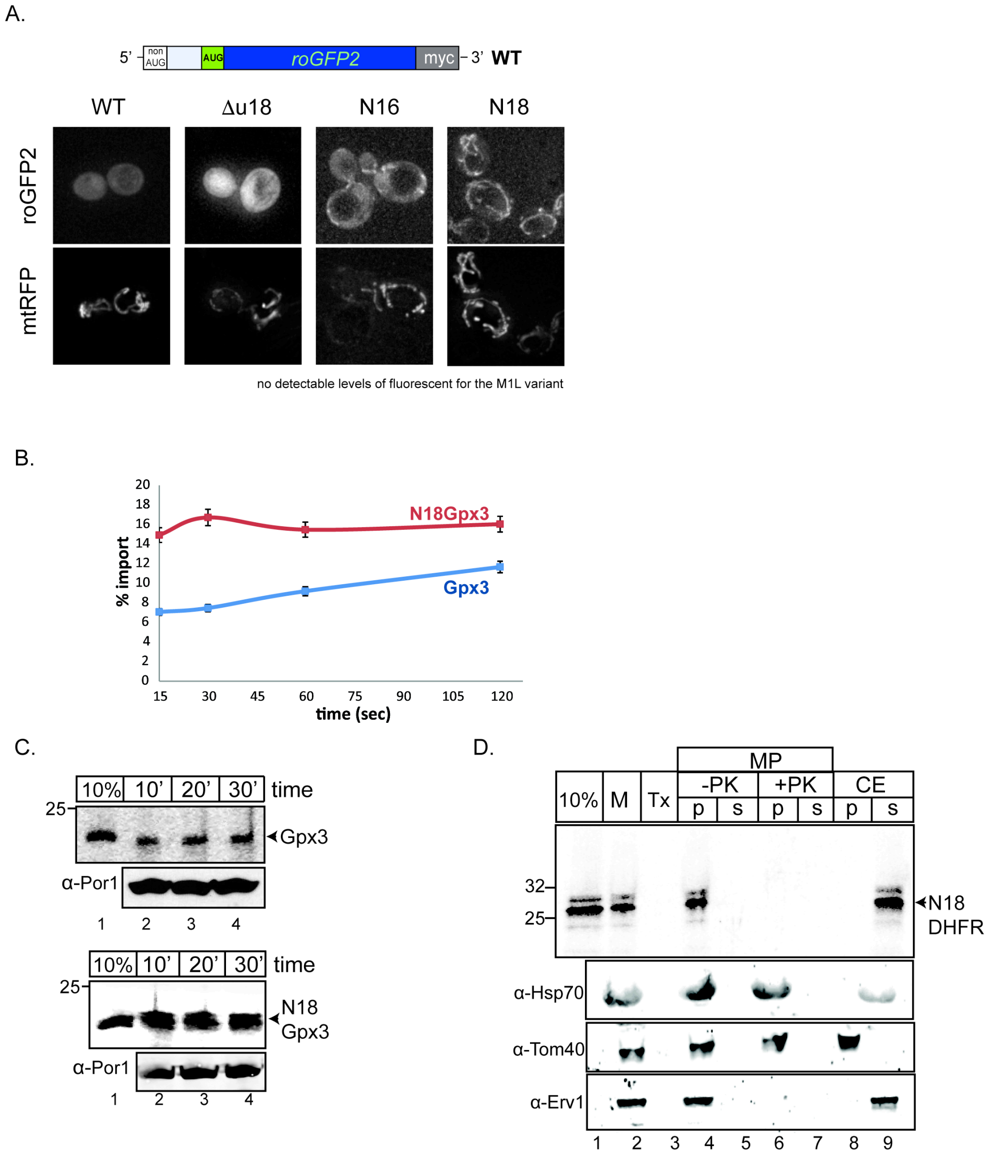

Figure S2, related to Figure 2: A. Gibson assembly was used to replace the *GPX3* sequence with roGFP2. BY4741 was transformed The epxression and localisation of the product was assessed using fluorescent microscopy. B. Kinetic analysis of the experiment presented in Figure 2F. C) Import of radiolabelled Gpx3 and N18Gpx3 in WT mitochondria at later time points (autoradiography). Equal loading was verified using the known mitochondrial marker protein, porin (αPor1). D) Import of radiolabelled N18DHFR in wild-type yeast mitochondria for 20min (M) (autoradiography). To verify the specificity of the import, samples were also treated with TritonX before exposure to protease (Tx). Further fractionation was additionally performed in a similar manner as in 1C, using osmotic shock in the presence or absence of protease (MP samples). Finally the mitochondrial soluble proteins were also obtained by carbonate extraction (CE). As in C, antibodies for known localised mitochondrial proteins were used. The 10% sample corresponds to the precursor that was used for the import reaction per 50µg of mitochondria (each lane).

A.

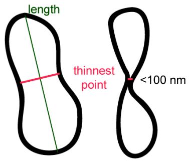

	WT	*gpx3*	*yap1*
Mitochondria examined	114	120	98
Thin mitochondria <100 nm (%)	8.7	29.1	6.1
Average thinnest point (nm)	187	154	191
Ratio length/thinnest point	1.89	2.49	1.92

B.

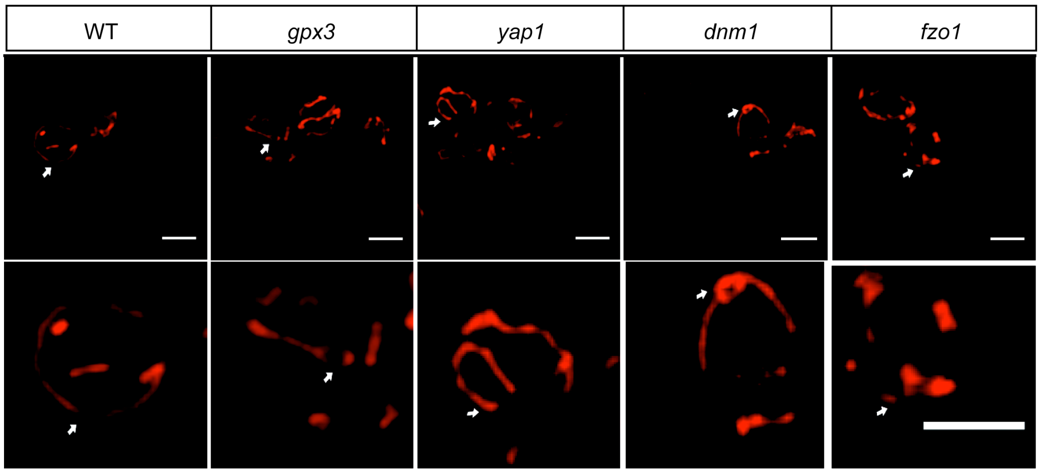

C.

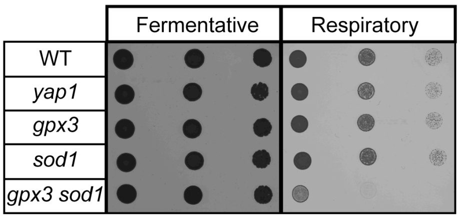

Figure S3, related to Figure 3: A.Quantitative analysis of ultrastructural parameters of EM images presented in Figure 3A.50 cells were randomly selected for each strain and properties of their mitochondria were examined. B. Assessement of mitochondrial morphology using fluorescent microscopy. WT,*gpx3*, *yap1*, *dnm1*, *fzo1* strains were transformed with a plasmid expressing mtRFP. Their mitochondrial morphology was monitored over time using microfluidic systems.Arrows indicate the ends of mitochondrial tubules. C. Spot test analysis of WT, *yap1*, *gpx3, sod1* and *gpx3 sod1* strain on fermentative (SCD) or respiratory (SCGE) media. Picture was taken after 2 days of growth.

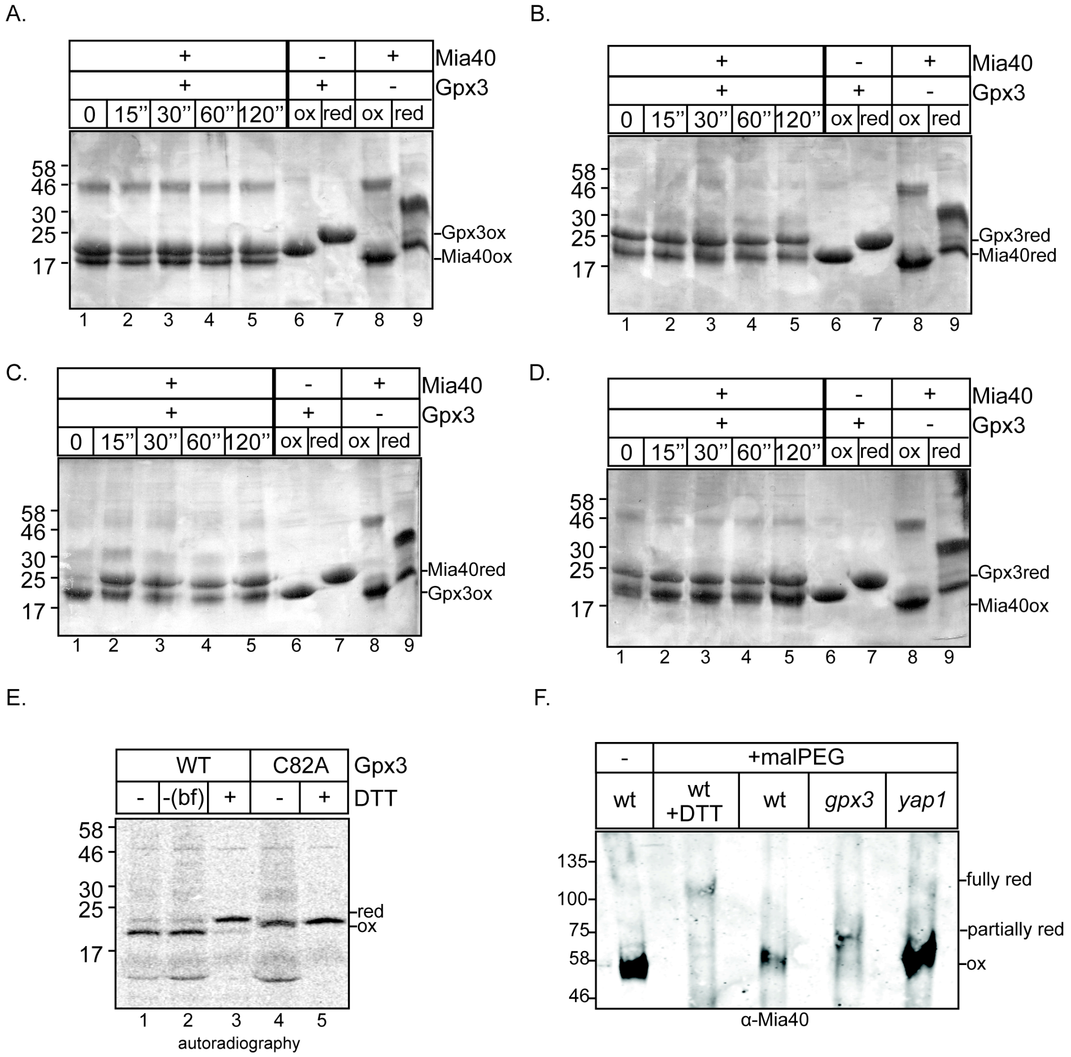

Figure S4, related to Figure 6: A-D) *In vitro* alkylation assays of all the possible redox-state couples of Gpx3 and Mia40. Several time points were taken to follow the progression of the reaction and follow changes in the redox state of the proteins via binding of the AMS agent. A) Gpx3 ox and Mia40 ox, B) Gpx3 red and Mia40 red, C) Gpx3 ox and Mia40 red, D) Gpx3 red and Mia40 ox. Only in panel C (Mia40 red and Gpx3 ox) we could see the appearance of the reduced form of Gpx3. E) Radiolabeled WT Gpx3 or C82A variant were treated with AMS as a control for the assays described in Figure 6B. F) Western blot analysis of the redox state of endogenous Mia40 in isolated wild-type, gpx3 and yap1 mitochondria that were blocked with TCA followed by malPEG labelling. wt+DTT idicate mitochondria that were DTT-treated before the malPEG labelling.

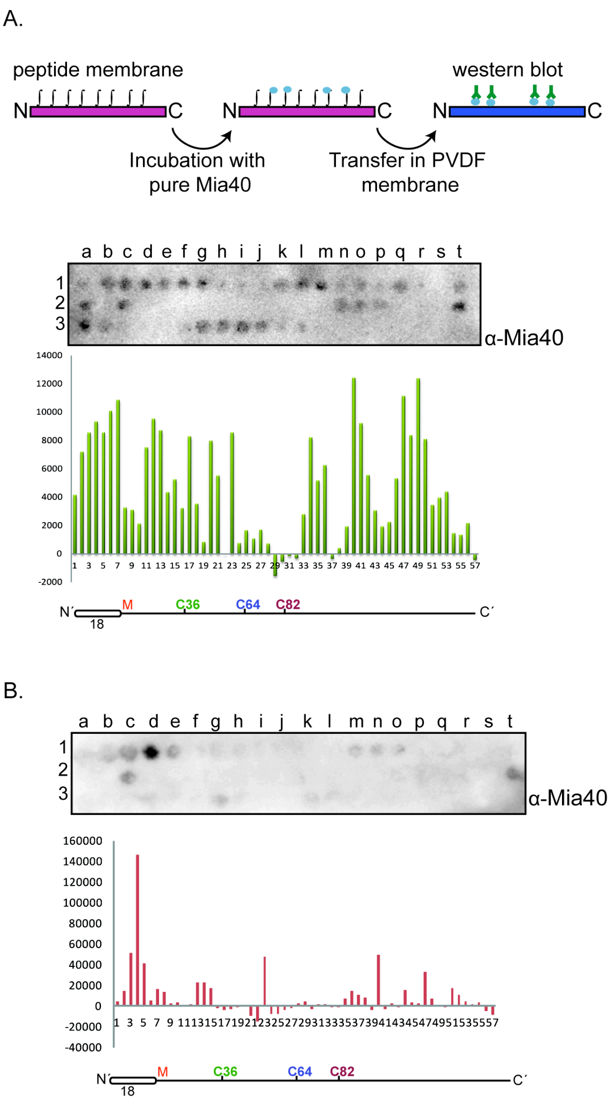

Figure S5, related to Figure 6**:** A) A peptide scan membrane, with immobilised 13-amino acid peptides,spanning the N18 Gpx3 sequence (overlap of 10aa) was incubated with purified ΔN290Mia40His. The binding was detected using antibodies against Mia40. The plot shows the possible regions where the two proteins bind and interact. B) To diminish the hydrophobic, non-covalent interactions we used the hydrophobic LMFFFM in a similar manner as in A.

Supplementary Experimental Procedures

Yeast strains and plasmids. BY4741 (MATa *his3Δ1 leu2Δ0 met15Δ0 ura3Δ0*) and its isogenic derivatives including *gpx3::KanMX4,* y*ap1::KanMX4, dnm1::KanMX4* and *fzo1::KanMX4* and *GPX3-GFP* were obtained from Life Technologies. Double mutant *gpx3 sod1* was generated by a cross of BY4741 *gpx3::KanMX4* and *BY4742 sod1::KanMX* and tetrad dissection. The Gpx3-myc plasmid contains 500bp of the promoter region of Gpx3 and the Gpx3 ORF followed by a triple myc-tag before the stop codon. The sequence was synthesised using GeneART and was then subcloned into a pRS415 vector using BamHI/EagI sites. To create the Gpx3 variants described in this study, we used site directed mutagenesis (Stratagene). Additionally, Gpx3 was subcloned in a pRS316-UP40-cytb2 plasmid that contains the Mia40 promoter and the targeting sequence of cytochrome b2 (N85). For the import experiments, Gpx3 and N18Gpx3 were subcloned in pSP64 vectors. All sequences were verified by sequencing (GATC biotech). The mtRFP plasmid was kindly provided by Campbell Gourlay (Leadsham et al., 2013; Weids and Grant, 2014). The roGFP2 constructs were generated using Gibson cloning (NEB). RoGFP2 was amplified from the roGFP2 plasmid that was a kind gift from Ian Dawes (Ayer et al., 2010).

Growth conditions. Strains were grown in SCD media [0.17% (w/v) yeast nitrogen base without amino acids, 5% (w/v) ammonium sulphate and 2% (w/v) glucose] or SCGE media [0.17% (w/v) yeast nitrogen base without amino acids, 5% (w/v) ammonium sulphate, 3% (v/v) glycerol and 1% (v/v) ethanol] supplemented with CSM complete amino acid mix or CSM –leu or –ura or –leu –ura mix (Formedium) at 30°C with agitation at 180rpm. Media were solidified with the addition of 2% (w/v) agar (Formedium). For spot tests, precultures were grown in SCR [0.17% (w/v) yeast nitrogen base without amino acids, 5% (w/v) ammonium sulphate and 2% (w/v)

raffinose] until stationary phase and dilutions (A_{600}= 1.0, 0.1, 0.01) were plated onto plates with different media. Growth was monitored after 2-3 days as indicated. For the complementation experiments, strains were transformed with plasmid variants and cells were grown on SD plates with CSM –leu or –ura (Formedium).

Cell breakage and western blotting. For western blot analysis, cells were broken in PBS pH 7.4 and EDTA-free protease inhibitors tablets (Roche) and 150µg of protein extract was loaded per lane. Gpx3-myc and variants were detected using α-myc 4A6 (Milipore) antibody. Anti-Pgk1 antibody was a kind gift from M. Tuite. Anti-mtHsp70 and anti-Porin antibodies were used as described previously (Greetham et al., 2013; Sideri et al., 2010; Sideris and Tokatlidis, 2007).

Immunoprecipitation assays. The import of radioactive N18Gpx3 and Gpx3His in wild-type mitochondria (15 min) was followed by removing the unimported material with trypsin (0.05 mg/ml). Mitochondria were then TCA precipitated to block electron transfer, washed with acetone and then resuspended in IP buffer (50 mM Tris pH 7.4, 50 mM NaCl, 5 mM EDTA and 2 mM PMSF). Dimedone (50mM) and H_2O_2 (100µM) were added for 30min at ice. Samples were then diluted with IP buffer and antibodies were added (2 hours, 4°C). The protein-A beads were blocked for 30min with 2 % BSA and then added to the reactions (1 hour, 4°C). The beads were washed with IP buffer prior to collection of the elution in Laemmli sample buffer. For the Mia40-Gpx3 interaction, mitochondria were solubilized in 0.16 % DDM for 30min at ice, followed by centrifugation at 15000g, 4°C, 30 min. The supernatant was diluted with IP buffer and antibodies were added. The rest of the procedure remained the same.

Purification of proteins and generation of antibodies For Gpx3 purification, the gene was cloned into the pET24, transformed into *E. coli* BL21 (DE3) cells. Protein

expression was induced with 0.4 mM IPTG for 4 hours at 37^oC. Cells were isolated and lysed in 300 mM NaCl, 50 mM Tris pH 7.4, 10 % glycerol, 20 μg/ml lysozyme using sonication. Sonicated samples were centrifuged at 21000g for 30 min at 4 ^oC and the supernatant was bound on Ni-NTA agarose beads. The protein was eluted with 300 mM imidazole in 50 mM Tris pH 7.4, 50 mM NaCl, 25 % glycerol. The protein was used for the production of antibodies against Gpx3, and also for *in vitro* interaction assays. The soluble recombinant Mia40, both the wild-type and the mutant, were expressed and isolated as described previously (Sideris et al., 2009).

Isolation of mitochondria. Isolation of yeast mitochondria was performed as described previously (Daum et al., 1982; Gasser et al., 1982). In brief, yeast cells were grown to an OD_{600} of 1 in YPLactate (unless stated otherwise). Cells were harvested by centrifugation at 2500g, and incubated in 0.1 M Tris-SO_4 pH 9.4, and 10 mM DTT (1.6 ml/g of cell pellet) for 20-25min at 30°C. Cells were pelleted and washed with 1.2 M sorbitol and 20 mM KPi, pH 7.4 (1ml/g of cell pellet). Next, cells were resuspended in 1.2 M sorbitol and 20 mM KPi, pH 7.4 (5ml/g of cell pellet) in the presence of Zymolyase (3.5 mg/g cell pellet) and incubated for 60min at 30°C. The spheroplasts were washed in the same buffer and homogenized on ice in 0.6 M sorbitol, 20 mM K-MES pH 6.0, 1 mM PMSF with a glass potter. Cell debris was removed by centrifugation at 1500g and mitochondrial-containing pellets were obtained by centrifuging supernatants at 12,000g. The pellets were homogenized using Teflon dounce, followed by a 1500g centrifugation to obtain the supernatant. The crude mitochondrial pellet was collected through a 12,000g spin for 15 min. Next, mitochondria were loaded on Nycodenz gradients (Nycodenz 20%-14.5%, 0.6 M sorbitol, 20 mM K-MES pH 6.0) followed by centrifugation (30 min, 25,000 rpm, 4^oC in a SW28 rotor). Pure mitochondria were collected and washed with 0.6 M sorbitol, 20 mM HEPES-KOH pH 7.4. Finally, the protein concentration was adjusted

to 20 mg/ml in the previous buffer by also adding 10 mg/ml BSA fatty-acid free. Mitochondria were frozen in liquid nitrogen and stored at −80°C.
