# Supplementary material for: Unconventional Targeting of a Thiol Peroxidase to the Mitochondrial Intermembrane Space Facilitates Oxidative Protein Folding
Source: Cell Rep. 2017 Mar 14;18(11):2729–41. doi: 10.1016/j.celrep.2017.02.053 (PMC5368413; doi:10.1016/j.celrep.2017.02.053)
Supplement: Document S2. Article plus Supplemental Information [file mmc2.pdf]

Cell Reports

Article

Unconventional Targeting of a Thiol Peroxidase to the Mitochondrial Intermembrane Space Facilitates Oxidative Protein Folding

Graphical Abstract

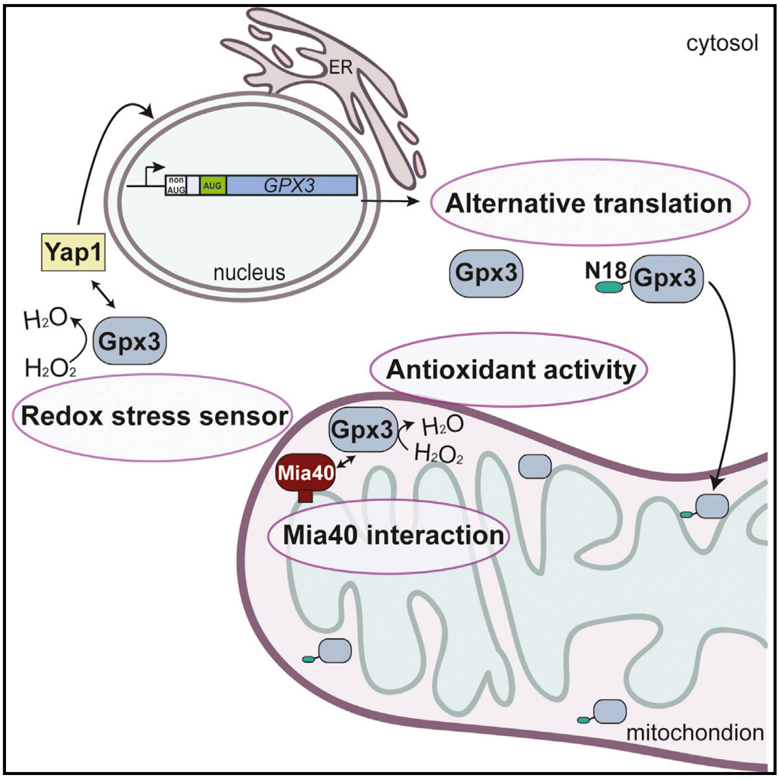

Highlights

- A pool of yeast Gpx3 localizes to mitochondria via translation from a non-AUG codon
- Loss of Gpx3 causes defects in mitochondrial architecture and membrane potential
- Gpx3 interacts with the oxidative protein folding machinery in the IMS

Authors

Paraskevi Kritsiligkou, Afroditi Chatzi, Georgia Charalampous, Aleksandr Mironov, Jr., Chris M. Grant, Kostas Tokatlidis

Correspondence

chris.grant@manchester.ac.uk (C.M.G.), kostas.tokatlidis@glasgow.ac.uk (K.T.)

In Brief

The redox sensor protein Gpx3 is imported into yeast mitochondria via a targeting sequence encoded from an upstream non-AUG codon. Kritsiligkou et al. show that mitochondrial Gpx3 acts in collaboration with the oxidative protein-folding machinery to ensure mitochondrial proteostasis and morphology.

CrossMark

Kritsiligkou et al., 2017, Cell Reports *18*, 2729–2741
March 14, 2017 © 2017 The Authors.
http://dx.doi.org/10.1016/j.celrep.2017.02.053

CellPress

Cell Reports
Article

OPEN ACCESS
CellPress

Unconventional Targeting of a Thiol Peroxidase to the Mitochondrial Intermembrane Space Facilitates Oxidative Protein Folding

Paraskevi Kritsiligkou,[1,3] Afroditi Chatzi,[2,3] Georgia Charalampous,[2] Aleksandr Mironov, Jr.,[1] Chris M. Grant,[1,*] and Kostas Tokatlidis[2,4,*]
[1]Faculty of Biology, Medicine and Health, University of Manchester, Manchester M13 9PL, UK
[2]Institute of Molecular, Cell and Systems Biology, College of Medical, Veterinary and Life Sciences, University of Glasgow, Glasgow G12 8QQ, UK
[3]Co-first author
[4]Lead Contact
*Correspondence: chris.grant@manchester.ac.uk (C.M.G.), kostas.tokatlidis@glasgow.ac.uk (K.T.)
http://dx.doi.org/10.1016/j.celrep.2017.02.053

SUMMARY

Thiol peroxidases are conserved hydrogen peroxide scavenging and signaling molecules that contain redox-active cysteine residues. We show here that Gpx3, the major H_2O_2 sensor in yeast, is present in the mitochondrial intermembrane space (IMS), where it serves a compartment-specific role in oxidative metabolism. The IMS-localized Gpx3 contains an 18-amino acid N-terminally extended form encoded from a non-AUG codon. This acts as a mitochondrial targeting signal in a pathway independent of the hitherto known IMS-import pathways. Mitochondrial Gpx3 interacts with the Mia40 oxidoreductase in a redox-dependent manner and promotes efficient Mia40-dependent oxidative protein folding. We show that cells lacking Gpx3 have aberrant mitochondrial morphology, defective protein import capacity, and lower inner membrane potential, all of which can be rescued by expression of a mitochondrial-only form of Gpx3. Together, our data reveal a novel role for Gpx3 in mitochondrial redox regulation and protein homeostasis.

INTRODUCTION

Disulfide bond formation is crucial for the native structure and stability of many proteins, while redox regulation through reversible cysteine oxidation is a common cellular strategy to adapt protein function to redox conditions. Conversely, oxidative stress may have detrimental effects on cell physiology through thiol oxidation, which is why cells have evolved several enzymatic mechanisms to cope with such conditions. These include the glutaredoxin and thioredoxin systems, which are the major cellular protein disulfide reduction systems (Morano et al., 2012).

Hydrogen peroxide (H_2O_2) is a reactive oxygen species (ROS) that can lead to oxidative damage but can also act as a signaling molecule (Veal and Day, 2011). It is normally produced within cells from the dismutation of superoxide anions, as a product of NADPH oxidases, or as a byproduct of the mitochondrial respiratory chain (Murphy, 2009). Other sources of H_2O_2 are the processes of fatty acid oxidation in peroxisomes and disulfide bond formation in the endoplasmic reticulum (ER) and the mitochondrial intermembrane space (IMS). In yeast cells, the signaling role of H_2O_2 is primarily mediated by the Gpx3 thiol peroxidase (Delaunay et al., 2002). H_2O_2 oxidizes Gpx3 in the cytosol, resulting in the formation of an intermolecular disulfide bond with Yap1, the transcription factor that regulates the hydroperoxide response. Subsequently, the active form of Yap1 is generated by the formation of intramolecular disulfide bridges within Yap1. In this manner, Gpx3 functions as a H_2O_2 transducer in the cytosol (Toledano et al., 2004).

Oxidative protein folding is temporally and spatially uncoupled from protein synthesis in the cytosol, where reduced glutathione and reductive enzymes inhibit the formation of disulfide bonds. Compartmentalization is therefore a critical part of the process. Furthermore, mitochondria are the main cellular source of ROS, and maintaining their redox balance is critical for the aging process and many age-related neurodegenerative diseases (Murphy, 2009). Mia40 shuttles disulfides to substrates in the IMS, functioning as a chaperone and inducing their folding. The flavin adenine dinucleotide (FAD)-sulfhydryl oxidase Erv1 generates disulfide bonds de novo using either molecular oxygen or cytochrome *c* and other proteins as terminal electron acceptors (Allen et al., 2005). A natural byproduct of this reaction is H_2O_2, and its level needs to be tightly controlled. In the ER, removal of H_2O_2 produced by the FAD-linked Ero1 is ensured by the peroxiredoxin PrxIV, which provides an efficient quenching system together with GPX7 and GPX8 (Tavender et al., 2010; Zito et al., 2010). So far, no such H_2O_2-sensing and/or removal system has been characterized for the mitochondrial IMS. However, it is apparent that there is a need to control the levels of H_2O_2 and the redox state of Mia40 and Erv1 in this compartment. Interestingly, a proteomic yeast mitochondrial analysis identified a list of cytosolic proteins as members of the IMS, including Gpx3 (Vögtle et al., 2012).

This is an open access article under the CC BY license (http://creativecommons.org/licenses/by/4.0/).

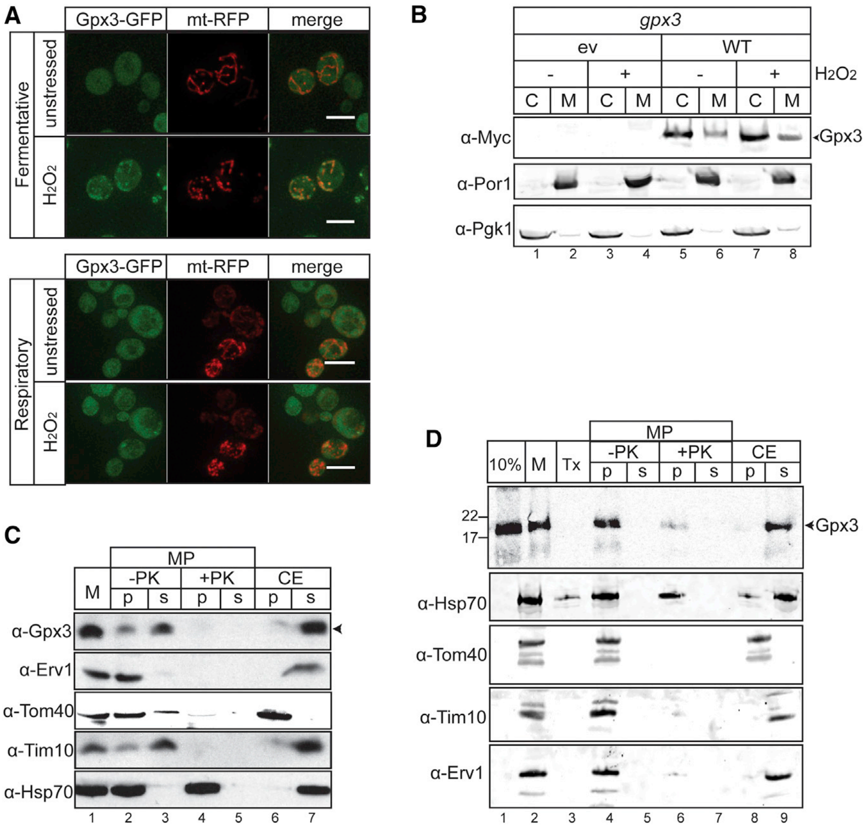

Figure 1. Gpx3 Is Localized in the Mitochondrial IMS

(A) A Gpx3-GFP genomically tagged strain was transformed with a plasmid expressing mtRFP. Cells were grown either in SCD (fermentative) or SCGE (respiratory) media and exposed to 1 mM H_2O_2 for 1 hr.

(B) A *gpx3* mutant strain was transformed with empty vector (ev) or a plasmid expressing Gpx3-myc (WT). Cells were grown in SD media until mid-exponential phase and treated with 1 mM H_2O_2. Cells were then fractionated to separate the cytosolic (C) and mitochondrial (M) fractions and western blots probed using antibodies against Gpx3-myc, mitochondrial porin (αPor1), and cytosolic Pgk1 (αPgk1).

(C) Fractionation of isolated WT yeast mitochondria (M). The outer mitochondrial membrane was removed with osmotic shock to create mitoplasts (MP) for protease (PK) access to the IMS. Soluble proteins (s) were obtained by carbonate extraction (CE), with the insoluble ones remaining in the pellet fraction (p). Western blots were probed with αErv1 and αTim10 for IMS proteins, αTom40 for the OM, and αHsp70 for the matrix.

(D) Import of radiolabeled Gpx3 in WT yeast mitochondria for 20 min (M) (autoradiography). To verify the specificity of the import, samples were also treated with Triton X before exposure to protease (Tx). Further fractionation was additionally performed in a similar manner as in (C), using osmotic shock in the presence or absence of protease (MP samples). Finally, the mitochondrial soluble proteins were also obtained by carbonate extraction (CE). The 10% sample corresponds to the precursor that was used for the import reaction.

Here, we characterized the role of Gpx3 in yeast mitochondria. We show that a fraction of Gpx3 is localized in the mitochondrial IMS in addition to the cytosol and that the mitochondrial form of Gpx3 is encoded by an upstream non-AUG codon that leads to an N-terminal extension capable of targeting proteins to the mitochondria. Furthermore, we show that cells lacking Gpx3 have aberrant mitochondrial morphology, display mitochondrial import defects, and lose their mitochondrial inner membrane potential upon H_2O_2-induced stress. The mitochondrial form of Gpx3 could rescue these phenotypes, suggesting a novel role for Gpx3 independent of its cytosolic function. Additionally, Gpx3 was shown to interact with Mia40 and maintain its redox state in vivo. Collectively, our data indicate that the mitochondrial IMS form of Gpx3 is linked to the oxidative folding machinery in this intracellular compartment.

RESULTS

Gpx3 Localizes to the Mitochondrial IMS

A previous proteomic analysis of the mitochondrial IMS identified Gpx3 (Vögtle et al., 2012). We validated the IMS localization of Gpx3 and examined whether it is altered in response to respiratory growth or oxidative stress conditions. Intracellular distribution was monitored using a Gpx3-GFP tagged version and a mitochondrially targeted RFP probe (mtRFP) for comparison (Leadsham et al., 2013). Gpx3 displayed strong cytoplasmic fluorescence under fermentative conditions (Figure 1A); this made it difficult to detect any mitochondrial localization. However, following H_2O_2-induced oxidative stress, mitochondria undergo fission, forming multiple fragmented structures (Figure 1A; H_2O_2). Under these conditions, we observed the co-localization of Gpx3 with mitochondria. Similarly, under respiratory conditions, Gpx3-GFP displayed predominantly cytoplasmic fluorescence, while co-localization with mitochondria was seen following H_2O_2 treatment (Figure 1A).

To further examine the mitochondrial localization of Gpx3, we used *gpx3* mutant cells expressing a functional myc-tagged version of Gpx3 under the control of its native promoter. We verified that Gpx3-myc rescued the sensitivity of a *gpx3* mutant strain to H_2O_2 (Figure S1A). Western blot analysis of mitochondrial and cytoplasmic fractions obtained by cell fractionation confirmed that a similar fraction of Gpx3 could be detected in mitochondria before and after H_2O_2 stress (Figure 1B). Additionally, we performed fractionation of wild-type mitochondria via osmotic shock and carbonate extraction (Figure 1C). Selective disruption of the outer membrane by hypotonic swelling to create mitoplasts (MP) followed by centrifugation releases the IMS content to the supernatant. Gpx3 was found in similar

amounts in both the pellet (associated with the inner membrane [IM]) and the supernatant IMS fraction (Figure 1C). Addition of Protease K (PK) during mitoplasting resulted in almost complete degradation of Gpx3 confirming its localization in the IMS. Furthermore, extraction by carbonate (CE) released the majority of Gpx3 into the supernatant, showing that Gpx3 is only weakly associated with the IM (Figure 1C).

We tested for import of Gpx3 in isolated wild-type mitochondria. ^{35}S-labeled Gpx3, produced using an in vitro reticulocyte translation system, was incubated with purified mitochondria and was found to be imported into a protease-protected location (Figure 1D). Solubilization of mitochondria with Triton X-100 (Tx) after import and subsequent addition of protease (PK) confirmed that Gpx3 could be cleaved and that import was specific to the mitochondria (Figure 1D). To assess the intra-mitochondrial localization of Gpx3, mitochondria were converted to mitoplasts. Gpx3 was found predominantly in the mitoplast pellet and was degraded in the presence of PK (Figure 1D), suggesting an association with the IM facing the IMS. Sodium carbonate extraction released most, but not all, Gpx3 into the supernatant, again suggesting an association with the IM (Figure 1D). Additionally, treatment of isolated yeast mitochondria with low urea did not further release Gpx3 into the supernatant, indicating that the IMS-localized Gpx3 has an affinity for the IM (Figure S1B). We also tested the cytosolic transcription factor Yap1 and the non-mitochondrial protein luciferase, confirming the import specificity under these conditions (Figure S1C). Taken together, these data indicate that a small but notable fraction of the cellular pool of Gpx3 localizes to the IMS of mitochondria and is associated with the outer surface of the inner mitochondrial membrane.

Gpx3 Is Imported into Mitochondria Using an N-Terminal Targeting Signal Encoded from a Non-AUG Codon

Examination of the N terminus of Gpx3 did not reveal any potential mitochondrial targeting sequences. However, a genome-wide ribosome profiling analysis of yeast grown under H_2O_2-induced stress identified a potential N-terminal extension within the *GPX3* mRNA, where translation is initiated upstream of the normal *GPX3* AUG start codon (Gerashchenko et al., 2012). We examined whether this N-terminal extension might encode a Gpx3 mitochondrial targeting sequence. While the study by Gerashchenko et al. (2012) identified ribosome binding upstream of the normal *GPX3* AUG start codon, the exact site of initiation was unclear, although potential non-AUG codons were observed (Michel et al., 2014). These would add an additional 18 amino acid N-terminal extension (encoded by a CTT codon 54 nt upstream of the normal start site) or a 16 amino acid N-terminal extension (encoded by an ACG codon 48 nt upstream of the normal start site). Using Mitoprot (prediction of mitochondrial targeting sequences for mitochondrial matrix proteins) (Claros and Vincens, 1996), both hypothetical N-terminal extensions of Gpx3 were predicted to encode targeting sequences with a high probability of mitochondrial import compared to wild-type Gpx3 (+18 amino acid [aa], 0.8744; +16 amino acid, 0.8058; WT, 0.0033).

To examine the effect of N-terminal extensions on the mitochondrial import of Gpx3, mutant versions were constructed using the wild-type Gpx3-myc plasmid as a template. In one construct, 54 nt upstream of the *GPX3* AUG codon were deleted to remove the entire putative mitochondrial targeting sequence (Figure 2A; Δu18). In a second construct, the *GPX3* AUG codon was removed by mutating it to a TTG codon (Figure 2A; M1L). We predicted that by removing the canonical *GPX3* AUG codon, we would be able to detect any longer forms of Gpx3 arising from non-AUG upstream translation initiation and that they would be targeted to mitochondria.

We examined the expression of plasmid-borne *GPX3* mutants in strains grown under fermentative growth conditions (Figure 2B) in the absence or presence of H_2O_2-stress using western blotting of total cell extracts. The Δu18 mutant was expressed at slightly lower levels than wild-type Gpx3 (Figure 2B). Importantly, a protein product was detected in the M1L mutant, indicating that translation initiation could still occur in the absence of the normal *GPX3* AUG codon (Figure 2B). H_2O_2-induced stress did not alter the production of any of the Gpx3 isoforms (Figure 2B).

Next, we examined the localization of Gpx3 mutants by isolating cytoplasmic and mitochondrial fractions (Figure 2C). As expected, wild-type Gpx3 was detected in both fractions (59% cytosolic and 41% mitochondrial) (Figure 2C). The Δu18 mutant was predominantly found in the cytosolic fraction (86%) (Figure 2C), whereas the M1L mutant was almost absent from the cytosolic fraction but detectable in the mitochondrial fraction (94%) (Figure 2C). To further confirm the potential role of an upstream sequence in the mitochondrial targeting of Gpx3, we introduced an AUG start codon 54 nt or 48 nt upstream of the position of the canonical start codon in the M1L mutant (Figure 2A). We reasoned that these constructs, named N18 (L(−18)M, M1L) and N16 (T(−16)M, M1L), would contain 18- and 16-amino-acid N-terminal extensions, respectively, which should promote mitochondrial localization. Both variants were expressed at significantly higher levels than the M1L mutant due to the presence of an AUG start codon (Figure 2D). Additionally, they predominantly localized to mitochondria, although cytosolic localization was still observed (Figure 2D). This is consistent with previous observations suggesting that the mitochondrial import machinery can become saturated; hence, the cytoplasmic form may represent Gpx3, which has not been processed and is trapped within the cytoplasm (Outten and Culotta, 2004).

We next questioned whether the N18 presequence is sufficient to drive a non-mitochondrial protein to mitochondria. An N18-DHFR construct was generated and examined in an import assay (Figure 2E). DHFR itself could not be imported, but the 18 amino acid extension acted as a targeting sequence leading to the efficient import of DHFR to mitochondria (Figure 2E). We also replaced the coding region of Gpx3 with GFP in the N18-Gpx3 and other mutant forms and followed their localization using fluorescence microscopy (Figures 2F and S2A). The N18-GFP construct localized to mitochondria, whereas the Δu18 variant did not (Figures 2F and S2A). Taken together, our data suggest that the 18 amino acid region upstream of the Gpx3 AUG start codon is sufficient to promote import of a non-targeted protein to mitochondria both in vivo and in organello.

We next assessed the effect of the N-terminal extension on targeting Gpx3 to mitochondria with import assays. Our own data (Figure 1D) and those of Vögtle et al. (2012) show that Gpx3 without an N-terminal extension can be imported into

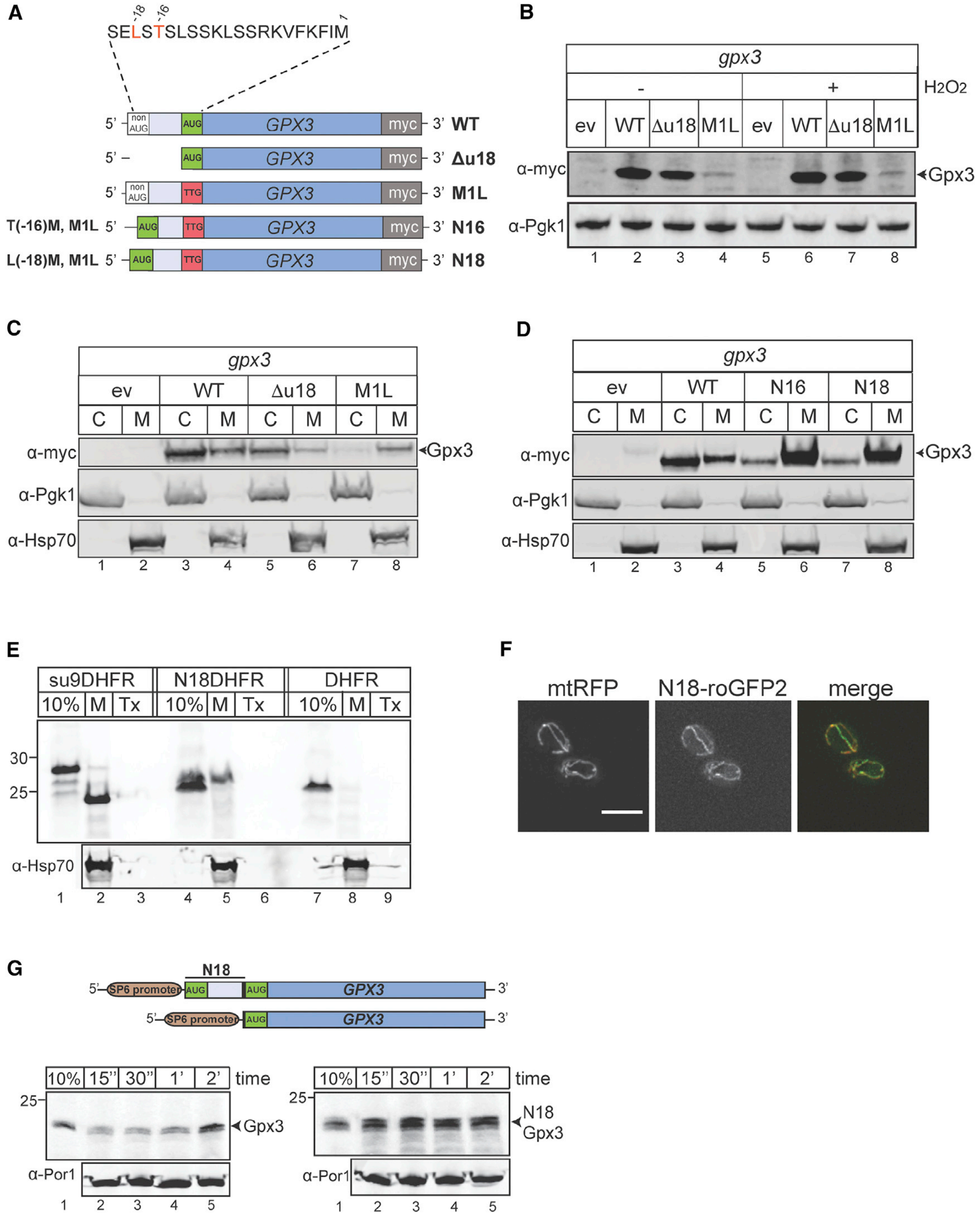

Figure 2. Gpx3 Is Localized to Mitochondria via an N-Terminal Extension Encoded from a Non-AUG Codon

(A) Schematic representation of Gpx3 variants. WT Gpx3-myc has its 500 bp 5′ UTR. Δu18 is lacking 54 nt prior to the AUG codon. M1L has the AUG mutated to TTG. N18 (L(−18)M, M1L) and N16 (T(−16)M, M1L) have AUGs introduced at positions −54 and −48, respectively, and the normal AUG start codon is mutated to TTG.

(B) Western blot analysis confirms protein expression from the Δu18 and M1L variants under fermentative growth and oxidative stress conditions. Cytosolic Pgk1 was used as a loading control.

(C and D) *gpx3* strains expressing ev, WT, Δu18, or M1L Gpx3 variants (C; depicted in A) were fractionated into cytosolic (C) and mitochondrial (M) fractions. *gpx3* strains expressing ev, WT, N16, or N18 variant (D; depicted in A) were fractionated into cytosolic (C) and mitochondrial (M) fractions. Western blots were probed against cytosolic Pgk1 and mitochondrial Hsp70 as controls.

(legend continued on next page)

OPEN
ACCESS
CellPress

isolated mitochondria. Localization experiments using radioactive N18 Gpx3 verified the presence of this longer version of Gpx3 in the IMS similar to wild-type Gpx3 (Figure S1D). Radioactive Gpx3 and N18 Gpx3 were imported into mitochondria at specific time points to compare their import capacity (Figure 2G). Both variants were imported efficiently, but early time point kinetics (15 s to 2 min) revealed that N18 Gpx3 displayed notably faster kinetics, peaking at 30 s with an import yield at least two times higher than Gpx3 (Figures 2G and S2B). At later time points, both variants were imported efficiently and at similar levels into isolated mitochondria; import appeared to plateau after 10 min (Figure S2C). These data indicate that the 18 amino acid region upstream of the normal Gpx3 AUG codon acts as a mitochondrial targeting sequence, likely affecting the early targeting events to the organelle.

Lack of Gpx3 Affects Mitochondrial Morphology and Function

Mitochondrial morphology defects are commonly observed as a result of mitochondrial dysfunction. To investigate the potential mitochondrial function of Gpx3, we examined the mitochondrial morphology of *gpx3* mutants using electron microscopy (Figure 3A). We also examined a *yap1* mutant to uncouple the putative function of Gpx3 in mitochondria from its established Yap1-linked cytoplasmic role. Comparing *gpx3* to wild-type yeast cells grown under fermentative conditions, we observed abnormalities in mitochondrial shape (Figure 3A). The most common feature was the presence of mitochondria in a distinctive "dumbbell" shape, as they appeared thicker at their ends while the middle section was thinner, stretched, and curved (Figure 3A). Quantification analysis confirmed the higher frequency of this mitochondrial abnormality in the *gpx3* strain (Figure S3A). To assess whether this morphological defect can be observed in vivo, we visualized mitochondria using fluorescent microscopy (Figure S3B). Again, the *gpx3* strain appeared to exhibit mitochondrial morphology similar to that observed using electron microscopy (EM) (Figures 3A and S3B).

Alterations in mitochondrial morphology are frequently associated with defects in the IM potential. We examined whether loss of *GPX3* abrogates the mitochondrial membrane potential in mitochondria isolated from wild-type and *gpx3* mutant strains by evaluating the incorporation of the fluorescent dye 3,3′-dipropylthiadicarbocyanine iodide ($DiSC_3(5)$) in mitochondria with active membrane potential (Figure 3B). The fluorescent signal is reduced as the dye becomes incorporated (addition of mitochondria to the reaction) and increases when the dye is released (addition of valinomycin to the reaction). We found that mitochondria from the *gpx3* strain exhibit a lower membrane potential (~25%) than wild-type mitochondria isolated under physiological conditions (Figure 3B).

As mitochondrial import into the IM and matrix requires the IM potential, we next examined the effect of the loss of *GPX3* on the import capacity of mitochondria using in vitro import experiments with isolated mitochondria. We used the matrix-targeted precursor Su9DHFR that has been widely used to study import into mitochondria (Allen et al., 2005; Geissler et al., 2000; Kurz et al., 1999; Sideris and Tokatlidis, 2007). We observed some minor differences between wild-type mitochondria and *gpx3* mitochondria prepared from cells grown under physiological conditions in the import of Su9DHFR, as the uncleaved precursor form of Su9DHFR was more pronounced in the *gpx3* mitochondria (Figure 3C). Additionally, in wild-type mitochondria isolated from H_2O_2-stressed cells, we observed the uncleaved precursor form, suggesting that the maturation of Su9DHFR was reduced (Figure 3C). When the precursor was imported in the *gpx3* mitochondria from H_2O_2-stressed cells, its maturation to the presequence-cleaved form was substantially abrogated, consistent with the previously observed defects in mitochondrial membrane potential (Figure 3C).

Hypothesizing that Gpx3 might act as an antioxidant in the mitochondrial IMS, we questioned whether a strain lacking Gpx3 might exhibit elevated levels of ROS in mitochondria. We used two approaches: (1) a genetic approach, where we deleted *SOD1*, a well-known IMS-localized antioxidant enzyme, and assessed the ability of the strains to grow on respiratory media (Figure S3C); and (2) an assay to directly measure the levels of mitochondrial ROS (Figure 3D). The genetic approach revealed that a *gpx3 sod1* strain grows poorly on respiratory media compared with the single-deletion strains. For the mitochondrial ROS measurements, we used MitoSOX, a fluorogenic dye that is targeted to mitochondria and becomes fluorescent upon oxidation by ROS exposure (Figure 3D). MitoSOX is thought to interact with superoxide, but it is also considered to be a useful indicator that can measure intracellular oxidant formation (Zielonka and Kalyanaraman, 2010). Higher fluorescence was detected in the *gpx3* mutant compared with the other strains, under normal growth conditions, indicating higher basal levels of oxidation in *gpx3* mitochondria (Figure 3D). The increased MitoSOX staining in a *gpx3* mutant (Figure 3D) could reflect a defective electron transport chain (ETC), leaking electrons and thus generating more superoxide. This interpretation is also consistent with the lower mitochondrial membrane potential (Figure 3B) and impaired import in *gpx3* mutants (Figure 3C).

Mitochondrial Gpx3 Rescues the Defects of a *gpx3* Deletion Strain

A strain lacking Gpx3 exhibits a wide variety of mitochondrial defects, but as Gpx3 is a signal transducer for Yap1 in the oxidative stress response, we examined whether mitochondrially localized Gpx3 is sufficient to rescue the mitochondrial phenotypes

(E) Import of radiolabeled precursors, Su9DHFR, DHFR, and N18DHFR in WT yeast mitochondria for 10 min. To verify the specificity of import, samples were treated with Triton X-100 before exposure to protease (Tx). Controls were similar to those used in Figure 1D.

(F) A WT strain expressing both an N18-roGFP2 plasmid and an mtRFP plasmid was grown to mid-exponential phase, and the localization of both fluorescent probes was examined with light microscopy.

(G) Schematic representation of the Gpx3 constructs used for the radioactive expression of the precursors in this study. Import of radiolabeled N18Gpx3 and Gpx3 in isolated WT yeast mitochondria for the indicated time points (autoradiography). Equal loading was verified using the known mitochondrial marker protein porin (αPor1). The 10% sample was used as a control, as in Figure 1D.

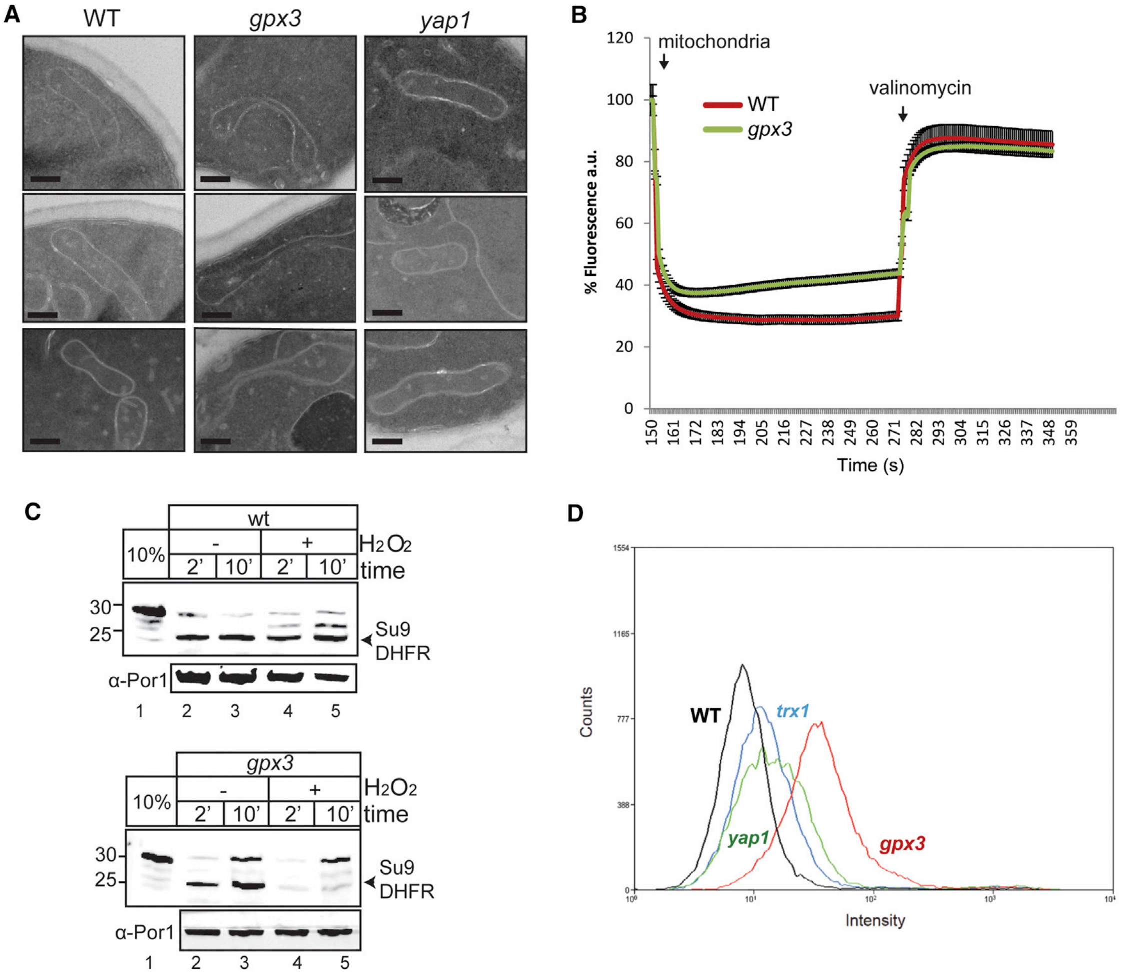

Figure 3. Strains Lacking Gpx3 Have Abnormal Mitochondrial Phenotypes

(A) EM analysis of mitochondria of WT, *gpx3*, and *yap1*. Scale bar, 200 nm. Representative examples are shown. Quantitative analysis is shown Figure S3A.

(B) WT and *gpx3* yeast mitochondria were incubated with $DISC_3(5)$ to assess the IM potential. Valinomycin was added to visualize the release of the dye from the mitochondria as a control. The signal of the dye in the reaction prior to the addition of mitochondria was set to 100%. Averages shown are from three repeats.

(C) Import of radioactive Su9DHFR in WT and *gpx3* yeast mitochondria for the indicated time points. Equal loading was verified using the mitochondrial marker protein αPor1. The 10% sample corresponds to the precursor that was used for the import.

(D) Staining for mitochondrial ROS was performed using MitoSOX. Representative histograms from three experimental repeats are presented.

observed. We investigated the import of Su9DHFR in mitochondria isolated from cells expressing different mutant forms of Gpx3 (Figure 4A). Mitochondria isolated from *gpx3* mutant cells transformed with a plasmid expressing wild-type Gpx3 (WT) or no Gpx3 (ev) showed import kinetics similar to those of WT and *gpx3* strains (Figures 3C and 4A). No major defects were observed for any *gpx3* mutant strains when the mitochondria were isolated from unstressed cells (Figure 4A). However, when mitochondria were isolated from H_2O_2-treated cells, differences in import capacity were observed. When mitochondria were isolated from cells containing either the M1L or N18 version of Gpx3, the maturation of Su9DHFR was partially rescued when compared to *gpx3*-depleted mitochondria (Figure 4A). In contrast, when isolated from cells expressing Δu18 and N16 Gpx3, the maturation pattern of Su9DHFR was similar to that observed in mitochondria lacking Gpx3 (Figure 4A). We also examined the Mia40-dependent, IMS-targeted protein Tim10 to evaluate whether import defects were only relevant for matrix-targeted proteins or whether loss of Gpx3 affects import pathways (like the MIA pathway) that do not depend on the membrane potential (Figure 4B). Import of Tim10 in *gpx3*-depleted mitochondria (Figure 4B) appeared to be less efficient than import into mitochondria from cells that contain Gpx3, independent of whether the cells were treated with H_2O_2 (Figure 4B). The import of Tim10 in mitochondria was restored to WT levels when any of the forms of Gpx3 were expressed in these cells (Figure 4B).

To examine whether the mitochondrial forms of Gpx3 can rescue mitochondrial morphology phenotypes, we used EM. We observed that mitochondria in *gpx3* strains transformed

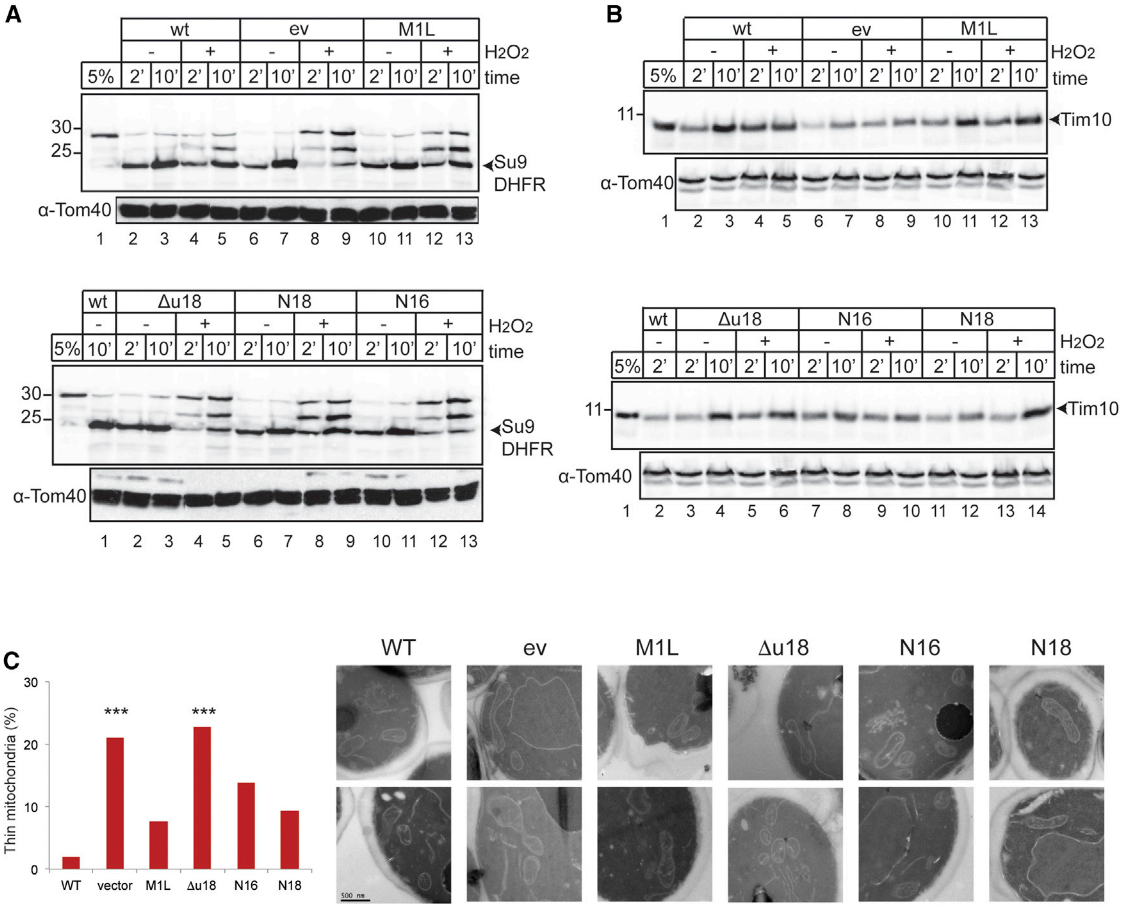

Figure 4. Mitochondrial Gpx3 Can Rescue Mitochondrial Defects of a *gpx3* Mutant

(A) Import of the radioactive precursor Su9DHFR in mitochondria isolated from *gpx3* strains expressing different forms of Gpx3 for the indicated time points. Equal loading was verified using antibodies against mitochondrial Tom40. The 5% sample corresponds to the precursor that was used for the import reaction.

(B) Same as (A), but the radioactive precursor of Tim10 was used instead of Su9DHFR.

(C) EM analysis of the same cells grown to mid exponential phase. Quantification of occurrence of thinner mitochondria is presented. Mitochondria from 50 random cells were quantified. Statistical analysis was performed using Fisher's exact test comparing the number of thinner mitochondria from the mutants to the WT form. ***$p < 0.001$.

with a plasmid encoding WT Gpx3 (WT) or the empty vector (ev) reflect the morphology of a WT and *gpx3* strain, respectively (Figures 3A and 4C). The M1L, N16, and N18 forms could rescue the abnormal morphology observed, whereas the Δu18 variant still had high levels of abnormal mitochondria, similar to the empty vector control (Figure 4C). Taken together, these data indicate that the mitochondrial import and morphology defects observed in a *gpx3* strain can be rescued when the mitochondrial, but not the cytosolic, form of Gpx3 is expressed, demonstrating a mitochondrial-specific role for Gpx3.

As both Δu18 and M1L appear to localize to the cytosol and mitochondria, albeit at different levels, we generated a variant of Gpx3 that only localizes to the mitochondrial IMS. We achieved this by generating a translational fusion of the cytochrome b2 (Cyb2) presequence that targets proteins to the IMS, upstream of Gpx3-myc, and is expressed under the control of the Mia40 promoter. Cyb2-Gpx3-myc is only found in the mitochondrial fraction, unlike the WT, which is dually localized in the cytosol and mitochondria (Figure 5A). The sub-mitochondrial localization of Cyb2-Gpx3 was assessed by mitoplasting, confirming that Cyb2-Gpx3 is localized in the mitochondrial IMS (Figure 5B). Having confirmed the IMS localization of Cyb2-Gpx3, we addressed whether an IMS-only form of Gpx3 could rescue any of the mitochondrial phenotypes of the *gpx3* mutant. First, we examined protein import and found that unlike mitochondria, where Gpx3 is absent and protein import of Su9-DHFR is impaired, mitochondria isolated from Cyb2-Gpx3-expressing strains have restored protein import capacity (Figure 5C). We then questioned whether this rescue occurs because Cyb2-Gpx3 restores the membrane potential defect of a *gpx3* strain and found that Cyb2-Gpx3 restores the membrane potential to levels similar to the WT (Figure 5D). In parallel, we examined whether the Cyb2-Gpx3 could act as an antioxidant in the IMS. Using viability assays, we found that Cyb2-Gpx3 can

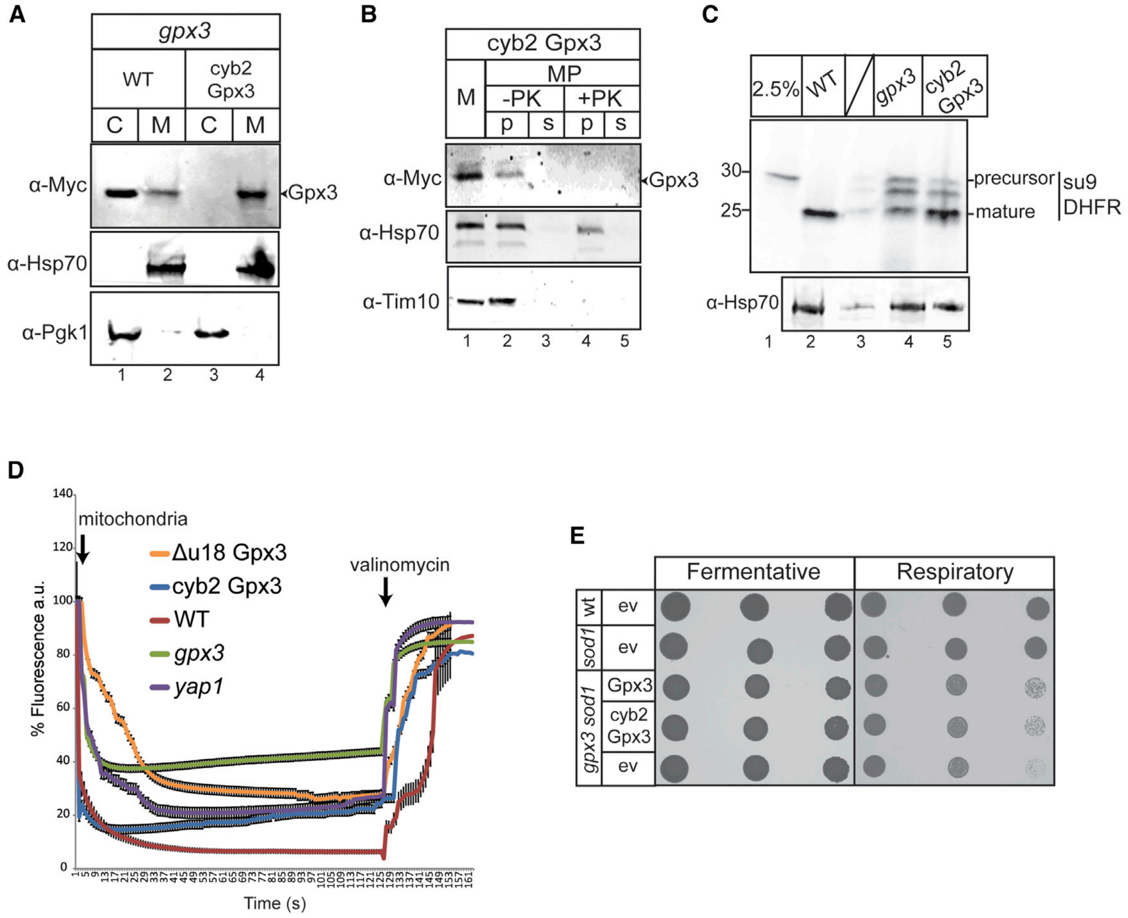

Figure 5. IMS-Targeted Gpx3 Rescues *gpx3* Phenotypes

(A) Fractionation of cells expressing cyb2 Gpx3 into cytosolic (‘C)’ and mitochondrial (‘M)’ fractions.

(B) Fractionation of isolated cyb2-Gpx3-Myc yeast mitochondria (M) as in Figure 1C.

(C) Import of radioactive precursor Su9DHFR in WT, *gpx3*, and cyb2 Gpx3 yeast isolated mitochondria for 15 min.

(D) As in Figure 3B, WT, *gpx3*, *yap1*, Δu18 Gpx3, and cyb2 Gpx3 yeast mitochondria were incubated with $DISC_3(5)$ to assess membrane potential. Error bars show the average of three repeats.

(E) Growth assays of WT and *sod1* cells expressing the empty vector and a *sod1 gpx3* strain expressing Gpx3, cyb2-Gpx3, or empty vector on fermentative or respiratory media. Cells were grown to stationary phase and dilutions of optical density (OD) 1, 0.1, and 0.01 were plated. Images were taken after 3 days of growth.

restore the growth of a *gpx3 sod1* mutant on respiratory media similar to WT levels (Figure 5E).

Gpx3 Can Oxidize Mia40

Given the role of Gpx3 in Yap1 oxidation, we asked whether Gpx3 has any similar redox-dependent interactions within the IMS. By drawing parallels with the ER, where PrxIV interacts with the PDI oxidoreductase (Tavender et al., 2010; Zito et al., 2010), we focused on the Mia40 mitochondrial oxidoreductase that is the functional counterpart of PDI in the mitochondrial oxidative folding machinery. We used an in vitro assay with purified proteins and followed changes in their redox state via alkylation (4-Acetamido-4’-maleimidylstilbene-2,2’-disulfonic acid [AMS] binding) to examine the possible interaction between Gpx3 and the soluble redox active core domain of Mia40 (called ΔN290Mia40His as in Lionaki et al., 2010; Sideris et al., 2009). We investigated the reaction between all four possible combinations of reduced and oxidized forms of the two proteins (Figures S4A–S4D). Interestingly, we only detected a change in their redox state when oxidized Gpx3 was mixed with reduced Mia40, suggesting a direct interaction and electron transfer between the two proteins (Figure S4C). This experiment is complicated, as oxidized Gpx3 and reduced Mia40 migrate similarly on SDS-PAGE gels. We repeated this experiment using western blot analysis and found that oxidized Gpx3 rapidly becomes reduced following incubation with reduced Mia40, concomitant with oxidation of Mia40 (Figure 6A).

We used an additional approach to examine the potential interaction of oxidized Gpx3 with reduced Mia40, where radiolabeled Gpx3 was incubated with purified ΔN290Mia40His. To confirm the AMS labeling result, we compared the migration of the reduced and oxidized form of Gpx3 to that of a cysteine

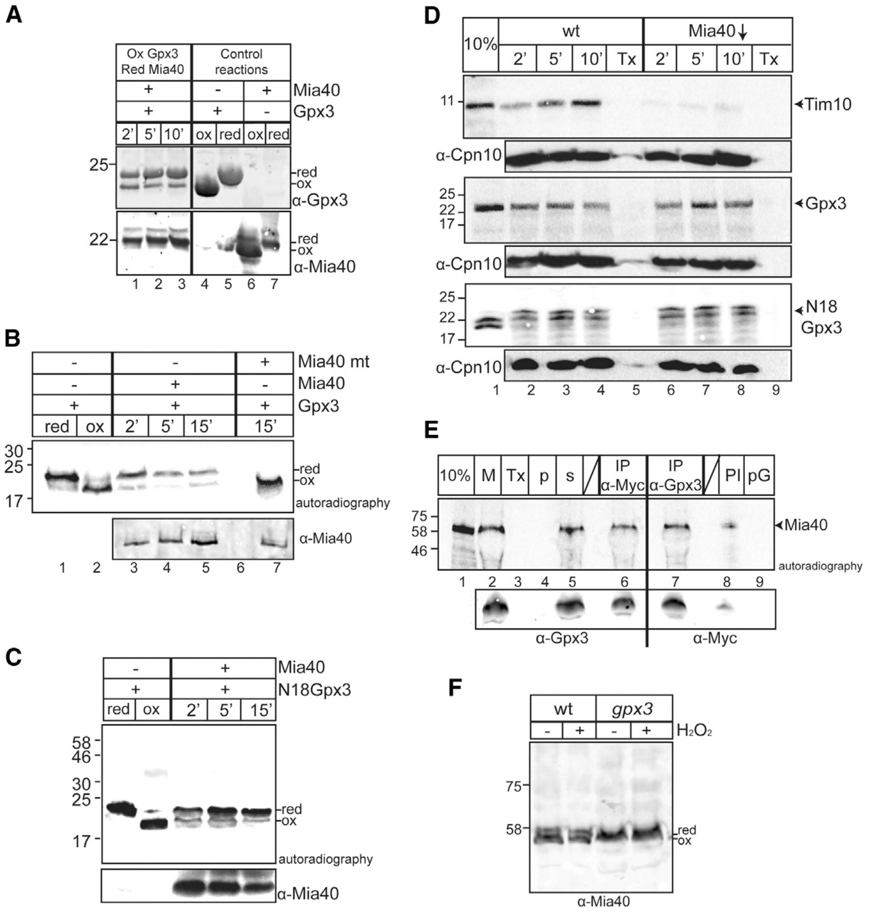

Figure 6. Gpx3 Interacts with Mia40 Both In Vitro and In Organello

(A) Oxidized recombinant Gpx3His and reduced ΔN290Mia40His (Mia40) were incubated in vitro for the indicated times. Reactions were stopped with TCA and followed by AMS labeling. Samples were visualized by western blot analysis.

(B) Same as in (A), except oxidized Gpx3His was radiolabeled and incubated with recombinant ΔN290Mia40His (Mia40) or the hydrophobic mutant (LMFFFM) of Mia40 (Mia40 mt). Samples were visualized by autoradiography.

(C) Oxidized N18 Gpx3 was incubated with reduced ΔN290Mia40His (Mia40) in an assay similar to (B).

(D) Import of radiolabeled Tim10, Gpx3, and N18Gpx3 in WT and Mia40-depleted yeast mitochondria for the indicated times. Samples were visualized with autoradiography prior to western blot analysis using mitochondrial Cpn10 for verification of equal loading.

(E) Radioactive SPCMia40 was imported in *gpx3*-Gpx3 mitochondria for 20 min (M). Mitochondria were then solubilized with 0.16% *n*-Dodecyl β-D-maltoside (DDM), and the supernatant (s) was separated from the pellet (p) and was incubated with either αMyc or αGpx3 for 2 hr to immunoprecipitate Gpx3Myc (IP samples). Reactions with pre-immune (PI) serum as well as protein beads alone (pA and pG) were used as a control. The 10% and Tx control samples were also loaded, as in Figure 6D. The immunoprecipitation of Gpx3 was done using both αMyc and αGpx3 antibodies.

(F) Western blot analysis of the redox state of endogenous Mia40 in isolated WT and *gpx3* mitochondria that were blocked with TCA followed by AMS labeling.

mutant of Gpx3 (Cys82Ala) (Figure S4E). Oxidized radiolabeled Gpx3 was incubated with reduced Mia40 and we observed a shift from the oxidized form of Gpx3 to the reduced form that occurs during the first 2 min of the reaction (Figure 6B). When an inactive mutant version of core Mia40 ΔN290Mia40His was used as a negative control, in which the hydrophobic LMFFFM motif is changed to alanine residues (Banci et al., 2009), no reduction of oxidized Gpx3 was observed (Figure 6B). To ensure that the N18 Gpx3 can also interact with Mia40, oxidized N18 Gpx3 was incubated with reduced Mia40 with similar results (Figure 6C).

A peptide scan array was used to independently confirm and map the putative interacting segments between Gpx3 and Mia40 (Figure S5). We used a membrane with immobilized 13 amino acid peptides, with an overlap of 10 amino acids, spanning the N18 Gpx3 sequence. This membrane was incubated with purified ΔN290Mia40His, and binding was detected using antibodies against Mia40 (Figure S5A). Interestingly, there are multiple regions of the Gpx3 sequence where Mia40 seems to bind preferentially. These are (1) the 18-amino-acid extension (residues 4–21) and (2) the C-terminal region (residues 139–150). The C-terminal region is rich in hydrophobic residues, reminiscent of the capacity of Mia40 to bind to protein partners through hydrophobic interactions (Banci et al., 2009; Milenkovic et al., 2009; Sideris et al., 2009). As a control, the Mia40 mutant, where the hydrophobic residues LMFFFM were all mutated to Ala to reduce hydrophobicity, showed a markedly diminished capacity to bind to N18 Gpx3 (Figure S5B).

We next confirmed the interaction between Gpx3 and Mia40 in organello. We first tested whether the import of Gpx3 is dependent on Mia40, as Mia40 is key to the import of many IMS proteins, particularly those with active cysteines (Kurz et al., 1999; Sideris and Tokatlidis, 2010a). Radioactive precursor Gpx3 and N18 Gpx3 were imported into isolated WT mitochondria and Mia40-depleted mitochondria derived from a strain where the expression of Mia40 is dependent on the presence of galactose in the media (Figure 6D). *MIA40* is an essential gene, and cells were grown on glucose-containing media to downregulate the expression of Mia40 prior to purification of these mitochondria. As a control, we examined the import of the MIA-dependent substrate Tim10, which was strongly reduced in the *MIA40*-depleted mutant (Figure 6D). Surprisingly, the import of both N18 Gpx3 and Gpx3 was largely unaffected in mitochondria lacking Mia40, in sharp contrast to Tim10. This suggests that Gpx3 is imported independently of the MIA machinery. Therefore, the observed in vitro interaction between Mia40 and Gpx3 therefore likely reflects an interaction of the two proteins post-import that occurs between the mature and folded proteins. To examine the

interaction between Mia40 and Gpx3 in organello, a radiolabeled cysteine trap mutant SPCMia40 was imported into mitochondria isolated from *gpx3* yeast cells expressing plasmid-borne Gpx3-Myc (Figure 6E). Mitochondria were then detergent-solubilized, and the extracts were incubated with antibodies against either Gpx3 or the Myc. Mia40 was immunoprecipitated in both cases, indicating a specific association of the two proteins in mitochondria (Figure 6E). As a control, mitochondrial extracts were incubated with pre-immune (PI) serum or with protein beads alone (pG) to verify the specificity of the binding (Figure 6E).

Finally, taking into consideration both the reduction in the import levels of Tim10 (Figure 4B) and the interaction between Mia40 and Gpx3 in organello (Figure 6E), we checked the redox state of Mia40 in *gpx3*-depleted mitochondria. Incubation of mitochondria with AMS revealed that in the absence of Gpx3, Mia40 is less oxidized than WT cells (Figure 6F). We further confirmed this by using MAL-PEG to label free thiols, as this provides greater shifts on a gel than AMS (Figure S4F). The alteration on the redox state of Mia40 could explain the import defects for Tim10 described above, as this precursor depends on the oxidized form of Mia40 to be imported.

DISCUSSION

An H_2O_2 detoxification system may be required in the IMS, since H_2O_2 is produced as a byproduct of the ETC and the Mia40-Erv1 oxidative protein-folding machinery (Daithankar et al., 2012). Our data suggest that mitochondrial Gpx3 may partially play this role. Gpx3 is targeted to the IMS via an alternative non-AUG translation initiation site located upstream of its canonical AUG codon that leads to an 18-amino-acid-long N-terminal extension sufficient to target any protein to the mitochondria. Translation from non-AUG codons in *S. cerevisiae* is well established (Zitomer et al., 1984), but paradigms of non-canonical translation that lead to alternative mitochondrial localization are very few, with the translation of tRNA synthetases being the best-characterized examples of such a process (Chang and Wang, 2004). This is not unique to yeast, as in higher eukaryotes, translation from non-AUG codons to target polypeptides to mitochondria has also been shown. Recent studies using ribosome footprinting have tried to elucidate translation initiation sites from non-AUG codons, and several proteins have been shown to have N-terminal extensions. One such example is PTEN, a phosphatase involved in the AKT signaling pathway that was shown to have an alternative translation initiation site that leads to a longer form called PTENα that is important for mitochondrial function (Liang et al., 2014).

Previous studies have highlighted the importance of redox regulation in mitochondrial import. For example, oxidative stress conditions block the import of proteins via the TIM22 pathway (Curran et al., 2004). Our data show that the loss of *GPX3* reduces import into the mitochondrial matrix, particularly during oxidative stress conditions. Other studies have shown the importance of redox systems for the import of mitochondrial proteins into the IMS. Loss of yeast thioredoxins leads to reduced import rates and accumulation of oxidized unimported pre-proteins (Durigon et al., 2012). However, the IMS localization of thioredoxin could provide an alternative explanation, since Trx1 may be responsible for maintaining the redox environment in the IMS, as well as providing the reducing power for Gpx3. Additionally in *S. pombe* thioredoxin reductase mutants (where thioredoxin would be oxidized), both Erv1 and Mia40 appear to be differentially oxidized (García-Santamarina et al., 2013).

Other studies have emphasized the importance of reductants in the IMS, such as glutathione and glutathione reductase, that can influence the redox state of Mia40 (Kojer et al., 2012, 2015). As there is no known glutathione reductase in the IMS, one can hypothesize that should the IMS become more oxidized, this could lead to several defects via inactivation of mitochondrial proteins. Recently, it has also been shown that oxidized glutathione has a role in determining mitochondrial fusion in HeLa cells (Shutt et al., 2012). Our observations are in agreement with these findings, as mitochondria from the *gpx3* mutant appear to exhibit fission defects and aberrant mitochondrial architecture. The fact that mitochondrial Gpx3 can rescue these phenotypes highlights the importance of the presence of Gpx3 in the mitochondria and its function in thiol regulation. Additionally, there are extensive overlaps between the thioredoxin and glutathione redox systems, and, for example, the thioredoxin system has been shown to reduce glutathione disulfide or oxidised glutathione (GSSG) (Tan et al., 2010). We propose that Gpx3 acts as a key thiol peroxidase in a previously unappreciated redox regulatory pathway in the IMS.

Peroxiredoxins have been implicated in oxidative protein folding in the ER, where PrxIV facilitates correct oxidative protein folding and plays a role in detoxifying H_2O_2 produced from the oxidative protein-folding machinery via Ero1 (Tavender and Bulleid, 2010). It was shown that PrxIV could rescue deletion phenotypes in the absence of Ero1 by oxidizing PDI (Tavender et al., 2010; Zito et al., 2010). Our data reveal a similar mechanism in mitochondria, as Gpx3 can interact with Mia40, an oxidoreductase, and oxidize its reduced form. These findings suggest that the role of peroxiredoxins in facilitating oxidative protein folding is highly conserved. It will be exciting to study in the future whether this new interaction between Gpx3 and Mia40 described here is related or independent from the well-known function of Erv1 in recycling reduced Mia40 back to its oxidized state during the mitochondrial oxidative folding process.

Thiol peroxidases are major antioxidants that provide an enzymatic defense against oxidative stress caused by hydroperoxides. Gpx3 is best classified as an atypical 2-Cys peroxiredoxin, as it forms an intramolecular disulfide bond as part of its catalytic cycle, which is reduced by thioredoxin (Delaunay et al., 2002). Here, we propose that apart from its essential function as a redox switch in the cytosol, Gpx3 functions as an oxidoreductase in the IMS (Figure 7). Therefore, loss of Gpx3 impacts the mitochondrial IM potential, maintenance of mitochondrial architecture, and mitochondrial protein import and folding via interaction with the Mia40 oxidative protein-folding machinery. Our data suggest that the role of Gpx3 in the IMS is different from its role as a redox switch in the cytosol, as the mitochondrial-only form of Gpx3 can rescue both the import defects and the abnormal mitochondrial morphology.

OPEN
ACCESS
CellPress

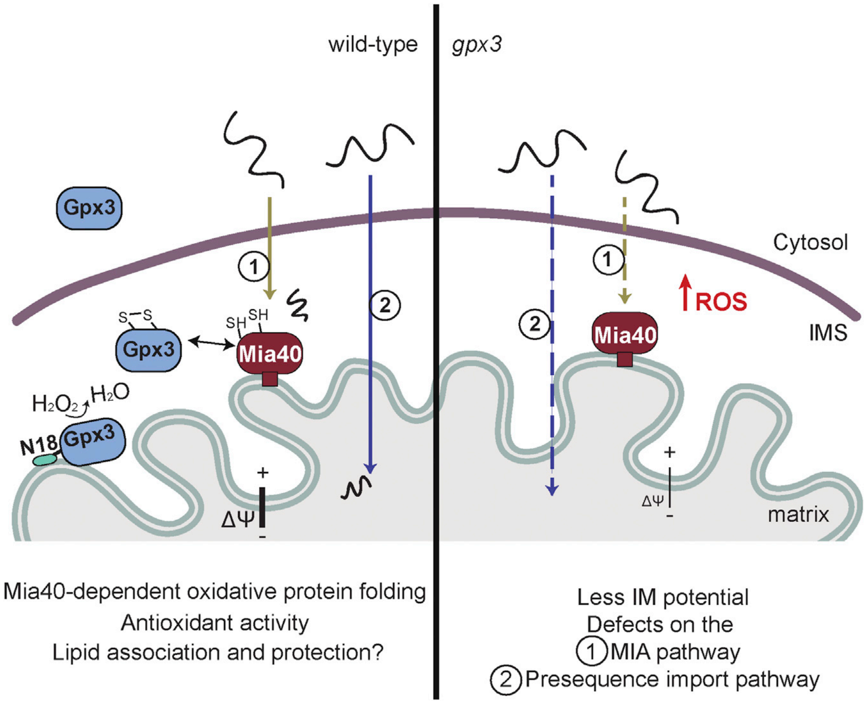

Figure 7. Model Depicting the Cellular Localization of the H_2O_2 Sensor Gpx3 and Its Role and Interactions in Mitochondria

Gpx3 is primarily found in the cytosol and in small amounts in the mitochondrial IMS. Both Gpx3 and N18 Gpx3 interact with Mia40, with a possible redox quality control role for the MIA pathway, and may serve an antioxidant function. Deletion of Gpx3 (shown in the right panel) results in elevated ROS levels, reduced mitochondrial IM potential, morphological anomalies, and defects in mitochondrial protein import pathways.

This study reveals an intriguing and unanticipated link between mitochondrial redox regulation and protein homeostasis, with important ramifications for oxidative metabolism and mitochondrial dysfunction.

EXPERIMENTAL PROCEDURES

Fluorescence Microscopy

A CellASIC microfluidic chamber was used to monitor live cells using a Delta Vision (Applied Precision) restoration microscope with a 100×/NA 1.40 Plan-Apo objective and fluorescein isothiocyanate (FITC) and Texas red band pass filters from the Sedat filter set (Chroma). The images were collected using a Coolsnap HQ (Photometrics) camera with a Z optical spacing of 0.25 μm. The raw images were deconvoluted using the Softworx software. All images were analyzed using ImageJ (https://imagej.nih.gov/ij).

EM Analysis

Samples were fixed with 4% formaldehyde and 2.5% glutaraldehyde in 0.1 M HEPES (pH 7.2). They were then infiltrated with 1% NaIO4 in water for 1 hr and stained with 1% uranyl acetate in water for 1 hr. The pellet was cut into small pieces, which were infiltrated with 1.6 M sucrose and 20% polyvinylpyrrolidone overnight, put on aluminum stubs, and frozen in liquid nitrogen. Ultrathin sections were cut with a Leica Ultracut UC6 ultramicrotome with FC6 cryo-chamber at −120°C and retrieved with the mix of 2.1 M sucrose and 2% methylcellulose. Sections were thawed, washed with 0.1M phosphate buffer, fixed with 1% Glutaraldehyde, and after washing with distilled water embedded into 1.8% methylcellulose with 0.5% uranyl acetate. Cells observed with an FEI Tecnai 12 Biotwin microscope at 100 kV accelerating voltage. Images were taken with Gatan Orius SC1000 charge-coupled device (CCD) camera.

Membrane Potential Measurement in Isolated Mitochondria

The membrane potential (Δψ) of isolated yeast mitochondria was assessed by measuring the fluorescence quenching of $DiSC_3(5)$ (Molecular Probes) as described previously (Gärtner et al., 1995; Sims et al., 1974). Measurements were performed using a Horiba JobinYvonFL-1039/40 Fluorimeter at 25°C (excitation 622 nm, emission 670 nm). The measurements were carried out in 1 mL 0.6 M sorbitol, 0.1% (w/v) BSA, 10 mM $MgCl_2$, 0.5 mM EDTA, and 20 mM KPi (pH 7.4). The following reagents were successively added, and the change in fluorescence was recorded: $DiSC_3(5)$ (in ethanol; final concentration, 2 μM); 50 μg mitochondria (in SEM buffer [250 mM sucrose, 1 mM EDTA, 10 mM MOPS-KOH, pH 7.2]) and, finally, valinomycin (in acetone; final concentration, 1 μM) to disrupt the potential. The difference in the fluorescence before and after the addition of valinomycin represents a relative assessment of the membrane potential. Each reaction was performed three times (individual aliquots of mitochondria), and then averages were calculated and presented as percentage values.

ROS Staining

For measurements using flow cytometry, cells were grown to mid exponential phase in SCD media and 10^5 cells collected by centrifugation. Cells were incubated in the dark with 2.5 μM MitoSOX (Life Technologies) for 30 min at 30°C. 30,000 cells from each experimental condition were analyzed using a flow cytometer (CyAn ADP, Beckman Coulter; excitation 488 nm, emission 520/30 nm).

Import in Yeast Mitochondria and Subfractionation of Mitochondria

^{35}S-labeled precursor proteins were synthesized using the TNT SP6-coupled transcription/translation kit (Promega) and plasmid-vectors pSP64 containing the genes of interest. The radioactive material was then denatured in 8 M urea, 50 mM HEPES (pH 7.4), 5 mM EDTA, and 20 mM DTT for 40 min at 30°C. The precursor is imported in 50 μg WT yeast mitochondria in the presence of 2 mM ATP and 2.5 mM NADH for the indicated time points at 30°C. Mitochondria were resuspended in 1.2 M sorbitol and 20 mM HEPES (pH 7.4), followed by a treatment with 0.05 mg/mL trypsin to remove unimported material for 30 min on ice (inactivation with 0.5 mg/mL soybean trypsin inhibitor [SBTI] for 10 min on ice). Mitoplasts were produced by resuspending mitochondria in 1× import buffer (Sideris and Tokatlidis, 2010b) at 5 mg/mL and dilution 10 times in 20 mM HEPES (pH 7.4) in the presence or absence of 0.1 mg/mL PK for 30 min on ice. The supernatant was kept for trichloroacetic acid (TCA) precipitation. For carbonate extraction, isolated mitochondria were resuspended in 0.1 M Na_2CO_3 and incubated on ice for 30 min; the pellet was then recovered by centrifugation (55,000 × *g*, 30 min, 4°C). Finally, samples were resuspended in Laemmli sample buffer with β-mercaptoethanol as indicated, analyzed by SDS-PAGE, and visualized by digital autoradiography (Molecular Dynamics). The quantification of the imported material based on the 10% samples was performed using the TotalLab Quant program.

In Vitro Interaction between Mia40 and Gpx3

Purified proteins were precipitated with ammonium sulfate and resuspended in 50 mM Tris (pH 8) with or without DTT (50 mM) to generate the oxidized or the reduced versions of these proteins. After 1 hr at room temperature (RT),

30 ng of each protein per reaction was incubated together for specific time points. Proteins were precipitated with 5% TCA, and AMS labeling was performed in buffer containing 50 mM Tris (pH 7.5), 3% w/v SDS, 3 mM EDTA, and 15 mM AMS for 30 min at 30°C and 30 min at 37°C (adapted from Mavridou et al., 2011). Proteins were run on non-reducing SDS-PAGE gels and visualized using Coomassie. The same procedure was followed with the radioactive precursor.

SUPPLEMENTAL INFORMATION

Supplemental Information includes Supplemental Experimental Procedures and five figures and can be found with this article online at http://dx.doi.org/10.1016/j.celrep.2017.02.053.

AUTHOR CONTRIBUTIONS

Conceptualization, P.K., A.C., C.M.G., and K.T.; Methodology, P.K., A.C., C.M.G., and K.T.; Investigation, P.K., A.C., G.C., A.M., C.M.G., and K.T.; Writing, P.K., A.C., G.C., A.M., C.M.G., and K.T.; Funding Acquisition, C.M.G., P.K., and K.T.; Supervision, C.M.G. and K.T.

ACKNOWLEDGMENTS

We would like to thank C. Gourlay (University of Kent) for mtRFP; P. March, S. Mardsen, and M.Jackson (University of Manchester) for help with microscopy and FACS; N. Katrakili (IMBB-FORTH, Crete) and D. McGow (University of Glasgow) for excellent technical assistance; and A.J. Weids (University of Manchester), R. Cogdell, and N. Bulleid (University of Glasgow) for critical reading and comments on the manuscript. The Bioimaging Facility microscopes used in this study were purchased with grants from BBSRC, the Wellcome Trust, and the University of Manchester Strategic Fund. This work was supported by a Wellcome Trust PhD studentship (096604/Z/11/Z) (P.K.), a BBSRC project grant (BB/M020770/1) (C.M.G.), the Wellcome Trust Institutional Strategic Support Funds to the University of Glasgow (097821/Z11/Z), the Scottish Universities Life Science Alliance and the Scottish Funding Council (HR07019), and the Royal Society-Wolfson research merit award (WM120111) (K.T.).

Received: July 27, 2016
Revised: November 1, 2016
Accepted: February 16, 2017
Published: March 14, 2017

REFERENCES

Allen, S., Balabanidou, V., Sideris, D.P., Lisowsky, T., and Tokatlidis, K. (2005). Erv1 mediates the Mia40-dependent protein import pathway and provides a functional link to the respiratory chain by shuttling electrons to cytochrome c. J. Mol. Biol. *353*, 937–944.

Banci, L., Bertini, I., Cefaro, C., Ciofi-Baffoni, S., Gallo, A., Martinelli, M., Sideris, D.P., Katrakili, N., and Tokatlidis, K. (2009). MIA40 is an oxidoreductase that catalyzes oxidative protein folding in mitochondria. Nat. Struct. Mol. Biol. *16*, 198–206.

Chang, K.J., and Wang, C.C. (2004). Translation initiation from a naturally occurring non-AUG codon in Saccharomyces cerevisiae. J. Biol. Chem. *279*, 13778–13785.

Claros, M.G., and Vincens, P. (1996). Computational method to predict mitochondrially imported proteins and their targeting sequences. Eur. J. Biochem. *241*, 779–786.

Curran, S.P., Leuenberger, D., Leverich, E.P., Hwang, D.K., Beverly, K.N., and Koehler, C.M. (2004). The role of Hot13p and redox chemistry in the mitochondrial TIM22 import pathway. J. Biol. Chem. *279*, 43744–43751.

Daithankar, V.N., Wang, W., Trujillo, J.R., and Thorpe, C. (2012). Flavin-linked Erv-family sulfhydryl oxidases release superoxide anion during catalytic turnover. Biochemistry *51*, 265–272.

Delaunay, A., Pflieger, D., Barrault, M.B., Vinh, J., and Toledano, M.B. (2002). A thiol peroxidase is an H2O2 receptor and redox-transducer in gene activation. Cell *111*, 471–481.

Durigon, R., Wang, Q., Ceh Pavia, E., Grant, C.M., and Lu, H. (2012). Cytosolic thioredoxin system facilitates the import of mitochondrial small Tim proteins. EMBO Rep. *13*, 916–922.

García-Santamarina, S., Boronat, S., Calvo, I.A., Rodríguez-Gabriel, M., Ayté, J., Molina, H., and Hidalgo, E. (2013). Is oxidized thioredoxin a major trigger for cysteine oxidation? Clues from a redox proteomics approach. Antioxid. Redox Signal. *18*, 1549–1556.

Gärtner, F., Voos, W., Querol, A., Miller, B.R., Craig, E.A., Cumsky, M.G., and Pfanner, N. (1995). Mitochondrial import of subunit Va of cytochrome c oxidase characterized with yeast mutants. J. Biol. Chem. *270*, 3788–3795.

Geissler, A., Krimmer, T., Bömer, U., Guiard, B., Rassow, J., and Pfanner, N. (2000). Membrane potential-driven protein import into mitochondria. The sorting sequence of cytochrome b(2) modulates the deltapsi-dependence of translocation of the matrix-targeting sequence. Mol. Biol. Cell *11*, 3977–3991.

Gerashchenko, M.V., Lobanov, A.V., and Gladyshev, V.N. (2012). Genome-wide ribosome profiling reveals complex translational regulation in response to oxidative stress. Proc. Natl. Acad. Sci. USA *109*, 17394–17399.

Kojer, K., Bien, M., Gangel, H., Morgan, B., Dick, T.P., and Riemer, J. (2012). Glutathione redox potential in the mitochondrial intermembrane space is linked to the cytosol and impacts the Mia40 redox state. EMBO J. *31*, 3169–3182.

Kojer, K., Peleh, V., Calabrese, G., Herrmann, J.M., and Riemer, J. (2015). Kinetic control by limiting glutaredoxin amounts enables thiol oxidation in the reducing mitochondrial intermembrane space. Mol. Biol. Cell *26*, 195–204.

Kurz, M., Martin, H., Rassow, J., Pfanner, N., and Ryan, M.T. (1999). Biogenesis of Tim proteins of the mitochondrial carrier import pathway: differential targeting mechanisms and crossing over with the main import pathway. Mol. Biol. Cell *10*, 2461–2474.

Leadsham, J.E., Sanders, G., Giannaki, S., Bastow, E.L., Hutton, R., Naeimi, W.R., Breitenbach, M., and Gourlay, C.W. (2013). Loss of cytochrome c oxidase promotes RAS-dependent ROS production from the ER resident NADPH oxidase, Yno1p, in yeast. Cell Metab. *18*, 279–286.

Liang, H., He, S., Yang, J., Jia, X., Wang, P., Chen, X., Zhang, Z., Zou, X., McNutt, M.A., Shen, W.H., and Yin, Y. (2014). PTENα, a PTEN isoform translated through alternative initiation, regulates mitochondrial function and energy metabolism. Cell Metab. *19*, 836–848.

Lionaki, E., Aivaliotis, M., Pozidis, C., and Tokatlidis, K. (2010). The N-terminal shuttle domain of Erv1 determines the affinity for Mia40 and mediates electron transfer to the catalytic Erv1 core in yeast mitochondria. Antioxid. Redox Signal. *13*, 1327–1339.

Mavridou, D.A., Saridakis, E., Kritsiligkou, P., Goddard, A.D., Stevens, J.M., Ferguson, S.J., and Redfield, C. (2011). Oxidation state-dependent protein-protein interactions in disulfide cascades. J. Biol. Chem. *286*, 24943–24956.

Michel, A.M., Fox, G., M Kiran, A., De Bo, C., O'Connor, P.B., Heaphy, S.M., Mullan, J.P., Donohue, C.A., Higgins, D.G., and Baranov, P.V. (2014). GWIPS-viz: development of a ribo-seq genome browser. Nucleic Acids Res. *42*, D859–D864.

Milenkovic, D., Ramming, T., Müller, J.M., Wenz, L.S., Gebert, N., Schulze-Specking, A., Stojanovski, D., Rospert, S., and Chacinska, A. (2009). Identification of the signal directing Tim9 and Tim10 into the intermembrane space of mitochondria. Mol. Biol. Cell *20*, 2530–2539.

Morano, K.A., Grant, C.M., and Moye-Rowley, W.S. (2012). The response to heat shock and oxidative stress in Saccharomyces cerevisiae. Genetics *190*, 1157–1195.

Murphy, M.P. (2009). How mitochondria produce reactive oxygen species. Biochem. J. *417*, 1–13.

Outten, C.E., and Culotta, V.C. (2004). Alternative start sites in the Saccharomyces cerevisiae GLR1 gene are responsible for mitochondrial and cytosolic isoforms of glutathione reductase. J. Biol. Chem. *279*, 7785–7791.

Shutt, T., Geoffrion, M., Milne, R., and McBride, H.M. (2012). The intracellular redox state is a core determinant of mitochondrial fusion. EMBO Rep. *13*, 909–915.

Sideris, D.P., and Tokatlidis, K. (2007). Oxidative folding of small Tims is mediated by site-specific docking onto Mia40 in the mitochondrial intermembrane space. Mol. Microbiol. *65*, 1360–1373.

Sideris, D.P., and Tokatlidis, K. (2010a). Oxidative protein folding in the mitochondrial intermembrane space. Antioxid. Redox Signal. *13*, 1189–1204.

Sideris, D.P., and Tokatlidis, K. (2010b). Trapping oxidative folding intermediates during translocation to the intermembrane space of mitochondria: in vivo and in vitro studies. Methods Mol. Biol. *619*, 411–423.

Sideris, D.P., Petrakis, N., Katrakili, N., Mikropoulou, D., Gallo, A., Ciofi-Baffoni, S., Banci, L., Bertini, I., and Tokatlidis, K. (2009). A novel intermembrane space-targeting signal docks cysteines onto Mia40 during mitochondrial oxidative folding. J. Cell Biol. *187*, 1007–1022.

Sims, P.J., Waggoner, A.S., Wang, C.H., and Hoffman, J.F. (1974). Studies on the mechanism by which cyanine dyes measure membrane potential in red blood cells and phosphatidylcholine vesicles. Biochemistry *13*, 3315–3330.

Tan, S.X., Greetham, D., Raeth, S., Grant, C.M., Dawes, I.W., and Perrone, G.G. (2010). The thioredoxin-thioredoxin reductase system can function in vivo as an alternative system to reduce oxidized glutathione in Saccharomyces cerevisiae. J. Biol. Chem. *285*, 6118–6126.

Tavender, T.J., and Bulleid, N.J. (2010). Peroxiredoxin IV protects cells from oxidative stress by removing H2O2 produced during disulphide formation. J. Cell Sci. *123*, 2672–2679.

Tavender, T.J., Springate, J.J., and Bulleid, N.J. (2010). Recycling of peroxiredoxin IV provides a novel pathway for disulphide formation in the endoplasmic reticulum. EMBO J. *29*, 4185–4197.

Toledano, M.B., Delaunay, A., Monceau, L., and Tacnet, F. (2004). Microbial H2O2 sensors as archetypical redox signaling modules. Trends Biochem. Sci. *29*, 351–357.

Veal, E., and Day, A. (2011). Hydrogen peroxide as a signaling molecule. Antioxid. Redox Signal. *15*, 147–151.

Vögtle, F.N., Burkhart, J.M., Rao, S., Gerbeth, C., Hinrichs, J., Martinou, J.C., Chacinska, A., Sickmann, A., Zahedi, R.P., and Meisinger, C. (2012). Intermembrane space proteome of yeast mitochondria. Mol. Cell. Proteomics *11*, 1840–1852.

Zielonka, J., and Kalyanaraman, B. (2010). Hydroethidine- and MitoSOX-derived red fluorescence is not a reliable indicator of intracellular superoxide formation: another inconvenient truth. Free Radic. Biol. Med. *48*, 983–1001.

Zito, E., Melo, E.P., Yang, Y., Wahlander, Å., Neubert, T.A., and Ron, D. (2010). Oxidative protein folding by an endoplasmic reticulum-localized peroxiredoxin. Mol. Cell *40*, 787–797.

Zitomer, R.S., Walthall, D.A., Rymond, B.C., and Hollenberg, C.P. (1984). Saccharomyces cerevisiae ribosomes recognize non-AUG initiation codons. Mol. Cell. Biol. *4*, 1191–1197.

Cell Reports, Volume *18*

Supplemental Information

Unconventional Targeting of a Thiol Peroxidase to the Mitochondrial Intermembrane Space Facilitates Oxidative Protein Folding

Paraskevi Kritsiligkou, Afroditi Chatzi, Georgia Charalampous, Aleksandr Mironov, Jr., Chris M. Grant, and Kostas Tokatlidis

Unconventional targeting of a thiol peroxidase to the mitochondrial intermembrane space facilitates oxidative protein folding

Paraskevi Kritsiligkou[1,3], Afroditi Chatzi[2,3], Georgia Charalampous[2], Aleksandr Mironov Jr.[1], Chris M. Grant[1,4], and Kostas Tokatlidis[2,4]

SUPPLEMENTARY MATERIAL

[1] Faculty of Biology, Medicine and Health, University of Manchester, Manchester, M13 9PL, UK

[2] Institute of Molecular, Cell and Systems Biology, College of Medical, Veterinary and Life Sciences, University of Glasgow, Glasgow, G12 8QQ, UK

[3] Co-first authors

[4] Co-senior authors

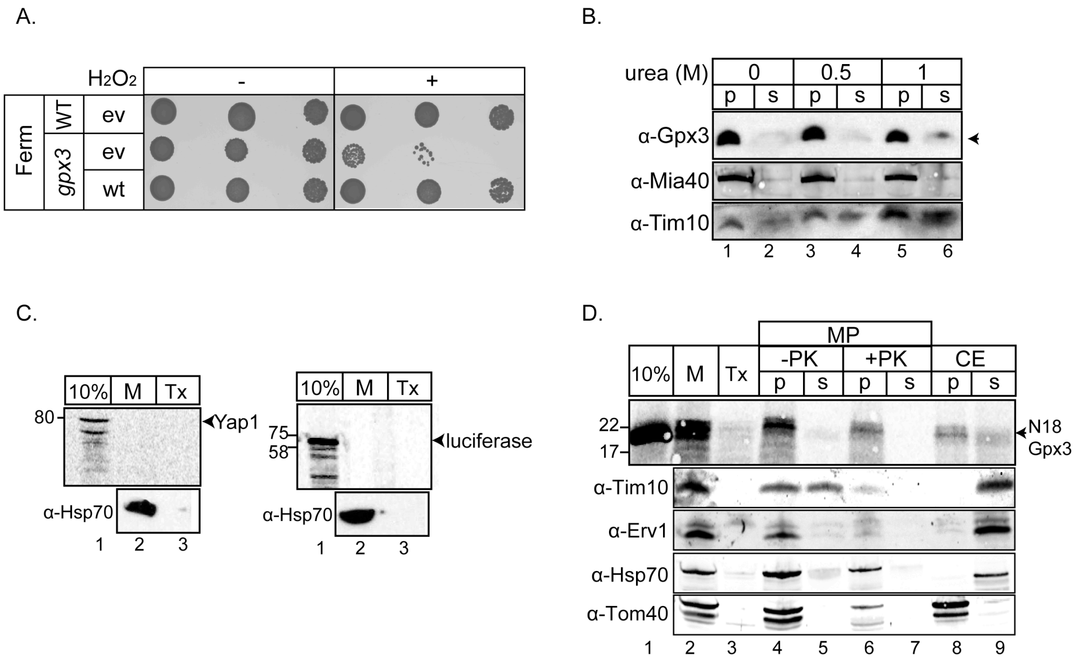

Figure S1, related to Figure 1: A) Gpx3 expressed myc-tagged from a plasmid can rescue the phenotype of a *gpx3* upon H2O2 stress similar to WT levels. Wt or *gpx3* strains were transformed with empty vector (ev) or WT (Gpx3-myc) plasmid. Cells were spotted on SCD (Ferm) plates with or without H_2O_2. B) WT mitochondria were incubated in SEH buffer (0.6 M sorbitol, 5 mM EDTA, 20 mM Hepes pH 7.4) containing different concentrations of urea for 10 min at 30°C. Mitochondria were isolated via centrifugation and the supernatant was TCA precipitated prior to SDS analysis and immunodecoration against proteins of interest. C) The radioactive precursors Yap1 and luciferase were prepared in the same conditions as Gpx3 and import assays in WT mitochondria were performed. D) Import of radiolabeled N18Gpx3 in WT mitochondria 20min (M) (autoradiography). To verify the specificity of the import, samples were also treated with TritonX before exposure to protease (Tx). Further fractionation was performed, using osmotic shock in the presence or absence of protease (MP samples). Finally the soluble proteins were also obtained by carbonate extraction (CE).

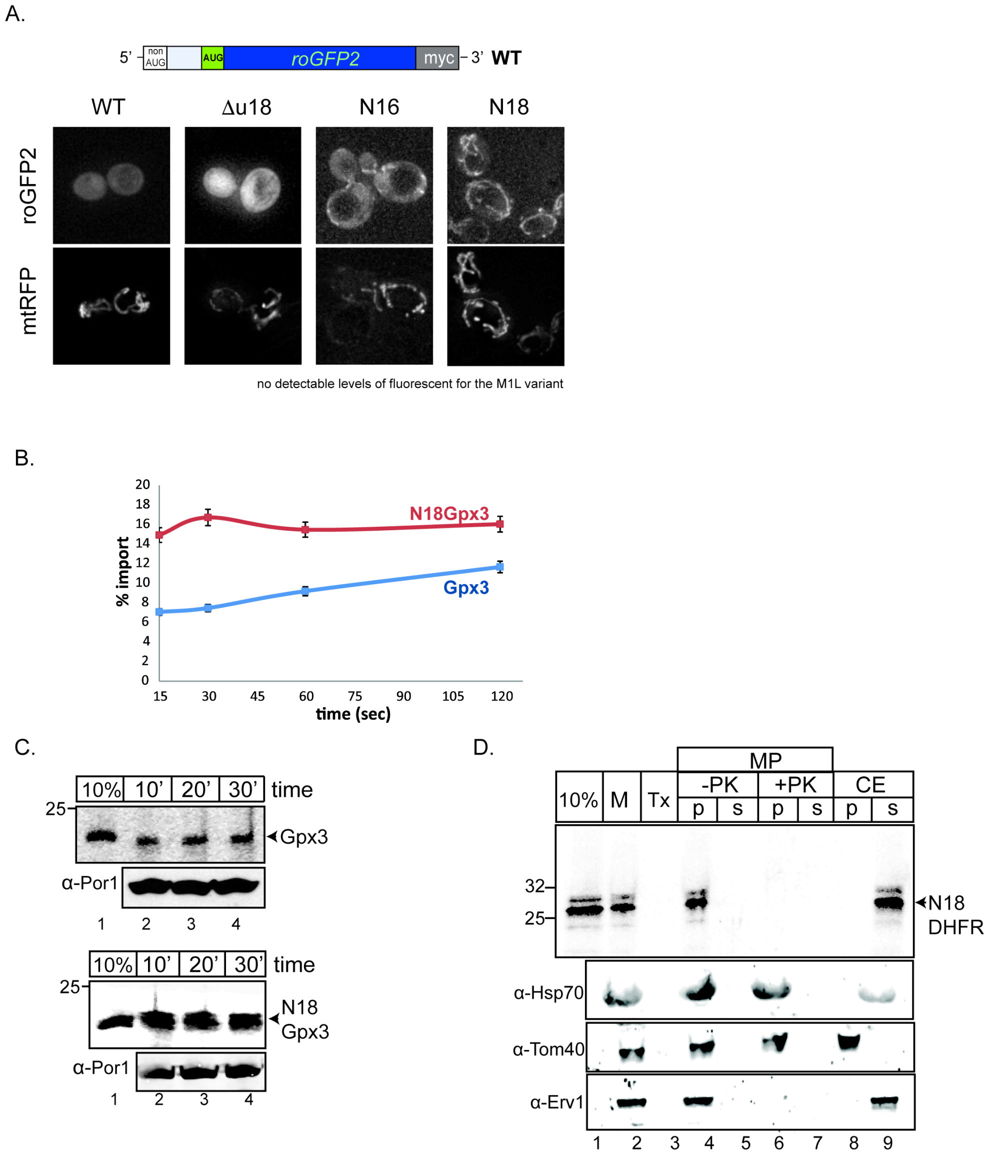

Figure S2, related to Figure 2: A. Gibson assembly was used to replace the *GPX3* sequence with roGFP2. BY4741 was transformed The epxression and localisation of the product was assessed using fluorescent microscopy. B. Kinetic analysis of the experiment presented in Figure 2F. C) Import of radiolabelled Gpx3 and N18Gpx3 in WT mitochondria at later time points (autoradiography). Equal loading was verified using the known mitochondrial marker protein, porin (αPor1). D) Import of radiolabelled N18DHFR in wild-type yeast mitochondria for 20min (M) (autoradiography). To verify the specificity of the import, samples were also treated with TritonX before exposure to protease (Tx). Further fractionation was additionally performed in a similar manner as in 1C, using osmotic shock in the presence or absence of protease (MP samples). Finally the mitochondrial soluble proteins were also obtained by carbonate extraction (CE). As in C, antibodies for known localised mitochondrial proteins were used. The 10% sample corresponds to the precursor that was used for the import reaction per 50μg of mitochondria (each lane).

A.

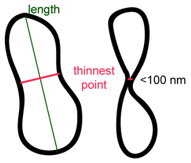

	WT	*gpx3*	*yap1*
Mitochondria examined	114	120	98
Thin mitochondria <100 nm (%)	8.7	29.1	6.1
Average thinnest point (nm)	187	154	191
Ratio length/thinnest point	1.89	2.49	1.92

B.

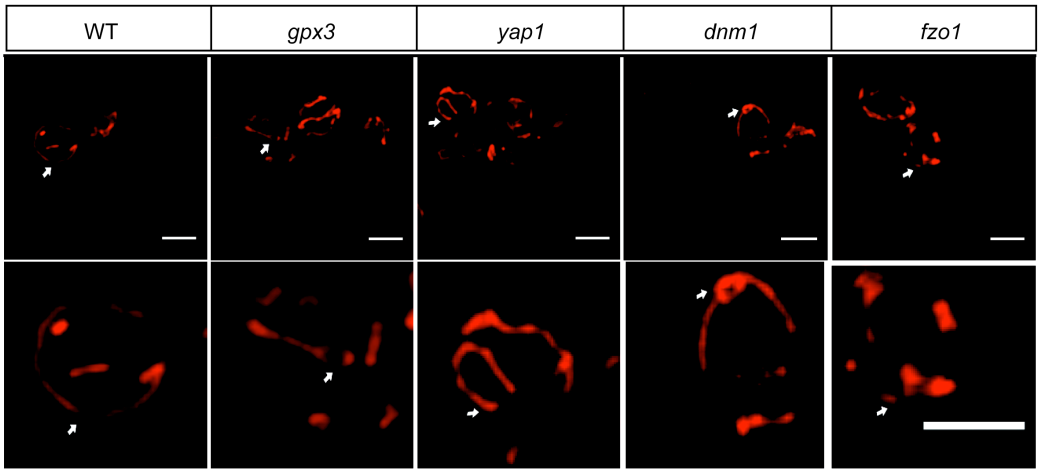

C.

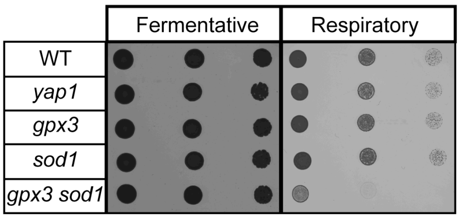

Figure S3, related to Figure 3: A.Quantitative analysis of ultrastructural parameters of EM images presented in Figure 3A.50 cells were randomly selected for each strain and properties of their mitochondria were examined. B. Assessement of mitochondrial morphology using fluorescent microscopy. WT,*gpx3*, *yap1*, *dnm1*, *fzo1* strains were transformed with a plasmid expressing mtRFP. Their mitochondrial morphology was monitored over time using microfluidic systems.Arrows indicate the ends of mitochondrial tubules. C. Spot test analysis of WT, *yap1*, *gpx3, sod1* and *gpx3 sod1* strain on fermentative (SCD) or respiratory (SCGE) media. Picture was taken after 2 days of growth.

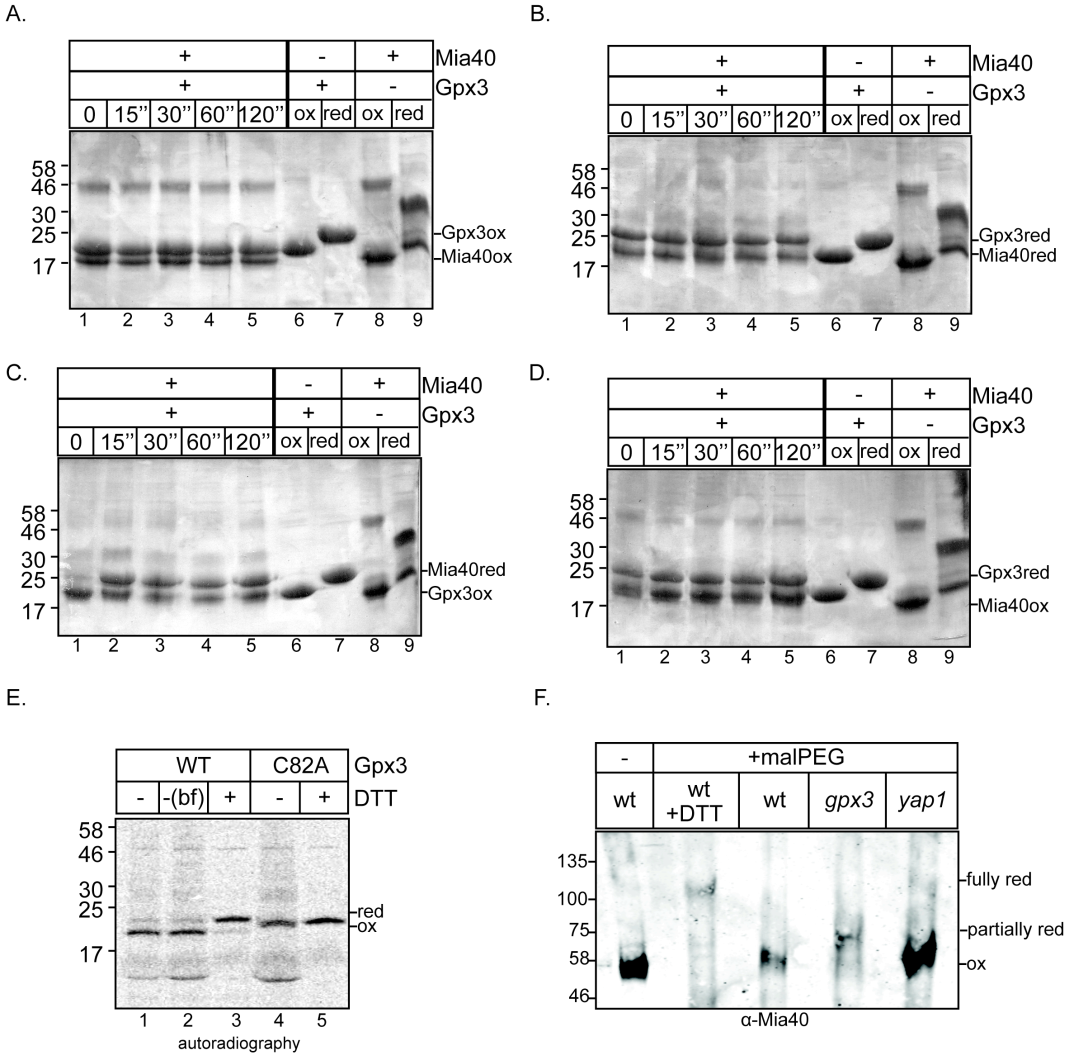

Figure S4, related to Figure 6: A-D) *In vitro* alkylation assays of all the possible redox-state couples of Gpx3 and Mia40. Several time points were taken to follow the progression of the reaction and follow changes in the redox state of the proteins via binding of the AMS agent. A) Gpx3 ox and Mia40 ox, B) Gpx3 red and Mia40 red, C) Gpx3 ox and Mia40 red, D) Gpx3 red and Mia40 ox. Only in panel C (Mia40 red and Gpx3 ox) we could see the appearance of the reduced form of Gpx3. E) Radiolabeled WT Gpx3 or C82A variant were treated with AMS as a control for the assays described in Figure 6B. F) Western blot analysis of the redox state of endogenous Mia40 in isolated wild-type, gpx3 and yap1 mitochondria that were blocked with TCA followed by malPEG labelling. wt+DTT idicate mitochondria that were DTT-treated before the malPEG labelling.

A.

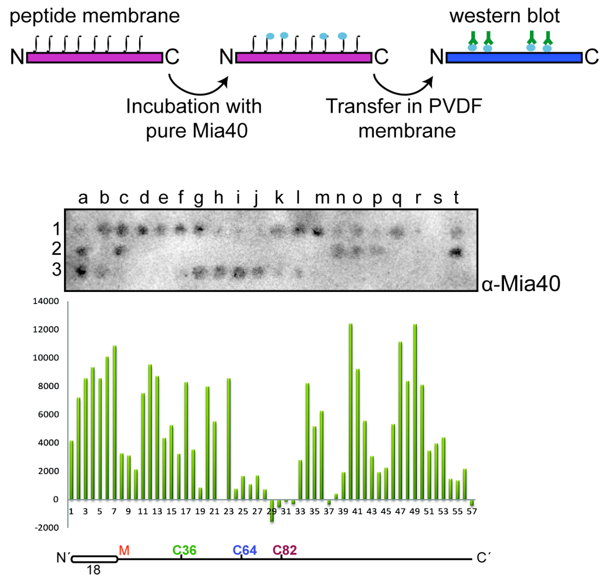

B.

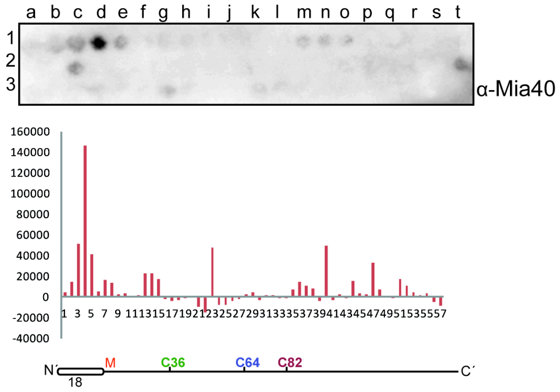

Figure S5, related to Figure 6**:** A) A peptide scan membrane, with immobilised 13-amino acid peptides,spanning the N18 Gpx3 sequence (overlap of 10aa) was incubated with purified ΔN290Mia40His. The binding was detected using antibodies against Mia40. The plot shows the possible regions where the two proteins bind and interact. B) To diminish the hydrophobic, non-covalent interactions we used the hydrophobic LMFFFM in a similar manner as in A.

Supplementary Experimental Procedures

Yeast strains and plasmids. BY4741 (MATa *his3Δ1 leu2Δ0 met15Δ0 ura3Δ0*) and its isogenic derivatives including *gpx3::KanMX4,* y*ap1::KanMX4, dnm1::KanMX4* and *fzo1::KanMX4* and *GPX3-GFP* were obtained from Life Technologies. Double mutant *gpx3 sod1* was generated by a cross of BY4741 *gpx3::KanMX4* and *BY4742 sod1::KanMX* and tetrad dissection. The Gpx3-myc plasmid contains 500bp of the promoter region of Gpx3 and the Gpx3 ORF followed by a triple myc-tag before the stop codon. The sequence was synthesised using GeneART and was then subcloned into a pRS415 vector using BamHI/EagI sites. To create the Gpx3 variants described in this study, we used site directed mutagenesis (Stratagene). Additionally, Gpx3 was subcloned in a pRS316-UP40-cytb2 plasmid that contains the Mia40 promoter and the targeting sequence of cytochrome b2 (N85). For the import experiments, Gpx3 and N18Gpx3 were subcloned in pSP64 vectors. All sequences were verified by sequencing (GATC biotech). The mtRFP plasmid was kindly provided by Campbell Gourlay (Leadsham et al., 2013; Weids and Grant, 2014). The roGFP2 constructs were generated using Gibson cloning (NEB). RoGFP2 was amplified from the roGFP2 plasmid that was a kind gift from Ian Dawes (Ayer et al., 2010).

Growth conditions. Strains were grown in SCD media [0.17% (w/v) yeast nitrogen base without amino acids, 5% (w/v) ammonium sulphate and 2% (w/v) glucose] or SCGE media [0.17% (w/v) yeast nitrogen base without amino acids, 5% (w/v) ammonium sulphate, 3% (v/v) glycerol and 1% (v/v) ethanol] supplemented with CSM complete amino acid mix or CSM –leu or –ura or –leu –ura mix (Formedium) at 30°C with agitation at 180rpm. Media were solidified with the addition of 2% (w/v) agar (Formedium). For spot tests, precultures were grown in SCR [0.17% (w/v) yeast nitrogen base without amino acids, 5% (w/v) ammonium sulphate and 2% (w/v)

raffinose] until stationary phase and dilutions (A_{600}= 1.0, 0.1, 0.01) were plated onto plates with different media. Growth was monitored after 2-3 days as indicated. For the complementation experiments, strains were transformed with plasmid variants and cells were grown on SD plates with CSM –leu or –ura (Formedium).

Cell breakage and western blotting. For western blot analysis, cells were broken in PBS pH 7.4 and EDTA-free protease inhibitors tablets (Roche) and 150μg of protein extract was loaded per lane. Gpx3-myc and variants were detected using α-myc 4A6 (Milipore) antibody. Anti-Pgk1 antibody was a kind gift from M. Tuite. Anti-mtHsp70 and anti-Porin antibodies were used as described previously (Greetham et al., 2013; Sideri et al., 2010; Sideris and Tokatlidis, 2007).

Immunoprecipitation assays. The import of radioactive N18Gpx3 and Gpx3His in wild-type mitochondria (15 min) was followed by removing the unimported material with trypsin (0.05 mg/ml). Mitochondria were then TCA precipitated to block electron transfer, washed with acetone and then resuspended in IP buffer (50 mM Tris pH 7.4, 50 mM NaCl, 5 mM EDTA and 2 mM PMSF). Dimedone (50mM) and H_2O_2 (100μM) were added for 30min at ice. Samples were then diluted with IP buffer and antibodies were added (2 hours, 4°C). The protein-A beads were blocked for 30min with 2 % BSA and then added to the reactions (1 hour, 4°C). The beads were washed with IP buffer prior to collection of the elution in Laemmli sample buffer. For the Mia40-Gpx3 interaction, mitochondria were solubilized in 0.16 % DDM for 30min at ice, followed by centrifugation at 15000g, 4°C, 30 min. The supernatant was diluted with IP buffer and antibodies were added. The rest of the procedure remained the same.

Purification of proteins and generation of antibodies For Gpx3 purification, the gene was cloned into the pET24, transformed into *E. coli* BL21 (DE3) cells. Protein

expression was induced with 0.4 mM IPTG for 4 hours at 37°C. Cells were isolated and lysed in 300 mM NaCl, 50 mM Tris pH 7.4, 10 % glycerol, 20 μg/ml lysozyme using sonication. Sonicated samples were centrifuged at 21000g for 30 min at 4 ^{o}C and the supernatant was bound on Ni-NTA agarose beads. The protein was eluted with 300 mM imidazole in 50 mM Tris pH 7.4, 50 mM NaCl, 25 % glycerol. The protein was used for the production of antibodies against Gpx3, and also for *in vitro* interaction assays. The soluble recombinant Mia40, both the wild-type and the mutant, were expressed and isolated as described previously (Sideris et al., 2009).

Isolation of mitochondria. Isolation of yeast mitochondria was performed as described previously (Daum et al., 1982; Gasser et al., 1982). In brief, yeast cells were grown to an OD_{600} of 1 in YPLactate (unless stated otherwise). Cells were harvested by centrifugation at 2500g, and incubated in 0.1 M Tris-SO_4 pH 9.4, and 10 mM DTT (1.6 ml/g of cell pellet) for 20-25min at 30°C. Cells were pelleted and washed with 1.2 M sorbitol and 20 mM KPi, pH 7.4 (1ml/g of cell pellet). Next, cells were resuspended in 1.2 M sorbitol and 20 mM KPi, pH 7.4 (5ml/g of cell pellet) in the presence of Zymolyase (3.5 mg/g cell pellet) and incubated for 60min at 30°C. The spheroplasts were washed in the same buffer and homogenized on ice in 0.6 M sorbitol, 20 mM K-MES pH 6.0, 1 mM PMSF with a glass potter. Cell debris was removed by centrifugation at 1500g and mitochondrial-containing pellets were obtained by centrifuging supernatants at 12,000g. The pellets were homogenized using Teflon dounce, followed by a 1500g centrifugation to obtain the supernatant. The crude mitochondrial pellet was collected through a 12,000g spin for 15 min. Next, mitochondria were loaded on Nycodenz gradients (Nycodenz 20%-14.5%, 0.6 M sorbitol, 20 mM K-MES pH 6.0) followed by centrifugation (30 min, 25,000 rpm, 4°C in a SW28 rotor). Pure mitochondria were collected and washed with 0.6 M sorbitol, 20 mM HEPES-KOH pH 7.4. Finally, the protein concentration was adjusted

to 20 mg/ml in the previous buffer by also adding 10 mg/ml BSA fatty-acid free. Mitochondria were frozen in liquid nitrogen and stored at −80°C.
